# The Role of Protected Areas in the Avoidance of Anthropogenic Conversion in a High Pressure Region: A Matching Method Analysis in the Core Region of the Brazilian Cerrado

**DOI:** 10.1371/journal.pone.0132582

**Published:** 2015-07-29

**Authors:** Rodrigo José Oliveira Paiva, Ricardo Seixas Brites, Ricardo Bomfim Machado

**Affiliations:** 1 Programa de Pós-Graduação em Geociências Aplicadas, Instituto de Geociências, Universidade de Brasília, Brazil; 2 Programa de Pós-Graduação em Zoologia, Departamento de Zoologia, Universidade de Brasília, Brazil; University of Sydney, AUSTRALIA

## Abstract

Global efforts to avoid anthropogenic conversion of natural habitat rely heavily on the establishment of protected areas. Studies that evaluate the effectiveness of these areas with a focus on preserving the natural habitat define effectiveness as a measure of the influence of protected areas on total avoided conversion. Changes in the estimated effectiveness are related to local and regional differences, evaluation methods, restriction categories that include the protected areas, and other characteristics. The overall objective of this study was to evaluate the effectiveness of protected areas to prevent the advance of the conversion of natural areas in the core region of the Brazil’s Cerrado Biome, taking into account the influence of the restriction degree, governmental sphere, time since the establishment of the protected area units, and the size of the area on the performance of protected areas. The evaluation was conducted using matching methods and took into account the following two fundamental issues: control of statistical biases caused by the influence of covariates on the likelihood of anthropogenic conversion and the non-randomness of the allocation of protected areas throughout the territory (spatial correlation effect) and the control of statistical bias caused by the influence of auto-correlation and leakage effect. Using a sample design that is not based on ways to control these biases may result in outcomes that underestimate or overestimate the effectiveness of those units. The matching method accounted for a bias reduction in 94–99% of the estimation of the average effect of protected areas on anthropogenic conversion and allowed us to obtain results with a reduced influence of the auto-correlation and leakage effects. Most protected areas had a positive influence on the maintenance of natural habitats, although wide variation in this effectiveness was dependent on the type, restriction, governmental sphere, size and age group of the unit.

## Introduction

The degradation of natural habitats in the tropical zone holds an important place on political agendas, both nationally and globally. For the purpose of containing threats to natural habitat areas, some environmental policy instruments, such as environmental certification and licensing, payment for ecosystem services [1], fiscal and commercial policies [2], and especially, the establishment of protected areas [3–5], have been employed for biodiversity conservation.

Although usually treated as a single strategy, protected areas have been established for different purposes, which were defined in the Convention of Biological Diversity (CBD) [6] as well as by national policy instruments. Most commonly, the purpose of these protected areas are to protect ecosystems and all their constituent species, protect ecosystem services, protect populations of specific threatened species, and even protect traditional cultures [1,7,8].

Given the variety of goals of these protected areas, studies on the effectiveness of those areas and their purposes have had different aims and subjects of analysis (e.g., effectiveness on improving park management, effectiveness on protected area system design, and effectiveness on conservation of specific species populations). The most common studies to date are analysis of the influence of protected areas on the preservation of natural habitats [9,10]. In this context, the effectiveness may be seen as a measure of the influence of protected areas (territories with special regulation of use and access) on avoidance of the anthropogenic conversion of the natural habitat. This attribute of protected areas may vary according to regional and local differences, methods used for evaluation [9,10], or even different restriction categories, which may vary from most restrictive to the least restrictive [11–14]. As a result, some studies point out the existence of units or categories of units, of which the effects are not different from those observed for non-protected regions [15–16]. Some studies even describe negative effects of certain protected areas with regard to habitat preservation [17–18].

Naughton-Treves et al. (2005) [3], Nagendra (2008) [7], and Geldmann et al. (2013) [9] conducted revisions of the studies on these protected areas and noticed that the studies unequally encompass different geographic or biogeographic regions, including unequal focus on different types of habitat. Of the 141 datasets described in the previous studies, a total of 132 were forest environments, while only three were savanna or shrubs/grassland habitats. Most of these types of studies performed in Brazil referenced the Amazon biome[19], while a few evaluated the Cerrado Biome [20].

The little attention given to non-forest environments, such as the Cerrado woodland savanna, is not consistent with the biological importance and the anthropogenic pressure on these regions. The Cerrado accounts for about 4% of the world’s biodiversity (S1 Table). The abundance of endemic species and the anthropogenic pressure make Cerrado one of the 34 world hotspots [21], which are areas globally recognized as of special interest to conservation. Despite the importance of the Cerrado for biodiversity conservation, this region has been especially threatened by agricultural business expansion and a government development policy that has been implemented in a non-integrated manner with the biological conservation policy. Recent changes in the Brazilian environmental legislation may aggravate this scenario, rendering the Cerrado as the biome with the biggest potential loss of natural coverage [22]. In addition, there have been ten occurrences of protected area downgrading, downsizing, and degazettement (PADDD), resulting in the reduction of 2,837 km^2^ of protected areas in this biome [23].

The latest evaluations on the preservation of Brazil’s natural habitat indicate that Cerrado is the biome with the largest absolute deforested area with a total of 982,227 deforested km^2^ [24], accounting for 49.16% of its original area of 2.03 million km^2^ [25]. This biome also has the highest current rate of deforestation among the Brazilian biomes [22], indicating that this is a more serious situation than even the Amazon biome, for example.

The conversion of a natural habitat into an anthropic region has direct consequences for ecological processes and patterns and is considered to be the biggest threat to world biodiversity [26–29]. This relationship occurs directly due to the reduction of natural habitat availability and indirectly due to changes in landscape attributes [30], climate change [28], and social changes that intensify the over-exploitation of environmental resources. These elements have a synergistic impact, resulting in a higher risk of biological population loss [27]. Because of its large area converted and environmental characteristics that are favorable for anthropic use, the savanna region is one of the regions that experiences higher risk for decreased biodiversity conservation [21].

The process of conversion of natural habitats in the savanna regions in Brazil has already induced considerable effects on its biota, driving Cerrado to the bottom of the list of six Brazilian biomes in terms of threatened species, behind only the Atlantic Forest biome [31,32]. In the near future, researchers anticipate changes in the spatial distribution of species and in the composition and main descriptors of biological communities due to the anthropic action concerning the use of the region and the soil and climate changes in the region [33,34]. A recent estimate indicated that between 6.4% and 8.4% of mammal species in the Cerrado biome will disappear by 2050 and that the biome’s central and southeast region will face the biggest losses in biodiversity [26].

Deforestation, either historical or current, is not evenly distributed throughout the different regions of Cerrado and is mainly concentrated in the southeast, south, and southwest regions. Due to the continental dimensions of Cerrado as well as its heterogeneity in terms of environment and biological composition, a concentrated distribution of deforestation in specific regions may indicate that species, communities, and biogeographic or physiographic units [35–37] typical to regions with a larger percentage of cleared area are at increased risk.

In the context of a threat to biodiversity, environmental services, and natural resources and in the presence of continuing conversion of Cerrado’s natural habitats, it is critical to determine whether public policies related to the creation of protected areas (units devoted primarily to the biodiversity protection and its attributes) are also efficient in reducing the anthropogenic conversion process on a regional scale and in a sub-regional scale as well as whether the effectiveness is dissimilar in different protected area subgroups.

Studies aimed at evaluating the effectiveness of protected areas in the region are especially challenging. This is due, in part, to great environmental heterogeneity and principally the large social, economic, demographic and historical differences that are observed among the sub-regions of the biome. All of these issues are determining factors for the occurrence of Land-Use and Cover-Change—LUCC events. One of the few studies done on the topic [20] addressed the effectiveness of protected areas for the entire Cerrado Biome, using matching methods and observing changes between the years 2002 and 2009, with predefined covariates whose effects were sought to be controlled. As a result, the authors demonstrated that, at a regional level, protected areas had a positive influence on the preservation of overall habitat, with differences being identified between strictly protected areas, multiple-use protected areas and Indigenous Lands. Like previous studies that were done on a regional scale [38], initiatives such as those conveyed in the above mentioned article are important for tracking general trends across large regions. However, for the Cerrado Biome, due to the large heterogeneity of factors that influence occurrences of deforestation, and the varying history, this approach can potentially be affected by other covariates that were not explicitly considered in the study. In addition, analysis that considers the Cerrado as a whole for a recent short range period may be impacted disproportionately by specific sub-regions. It is expected, therefore, that sub-regional analysis is especially promising for the Biome, reflecting more accurately the influence of protected area units on LUCC.

This study aims to evaluate the effect of those areas in preventing the progress of deforestation in natural areas of the Cerrado region, which can be used as a proxy for regions with high anthropogenic impact. In addition to an analyzing the degree of restriction for protected areas discussed in the study, we observed other features, such as government involvement, the size of the areas and time since creation, that potentially influence the effectiveness of these units on the region. This study also considers other, additional, areas that are included in the Brazilian government’s biodiversity conservation strategy, such as the Indigenous Lands and Quilombolas Lands.

## Materials and Methods

### Study Area

**Fig 1:**
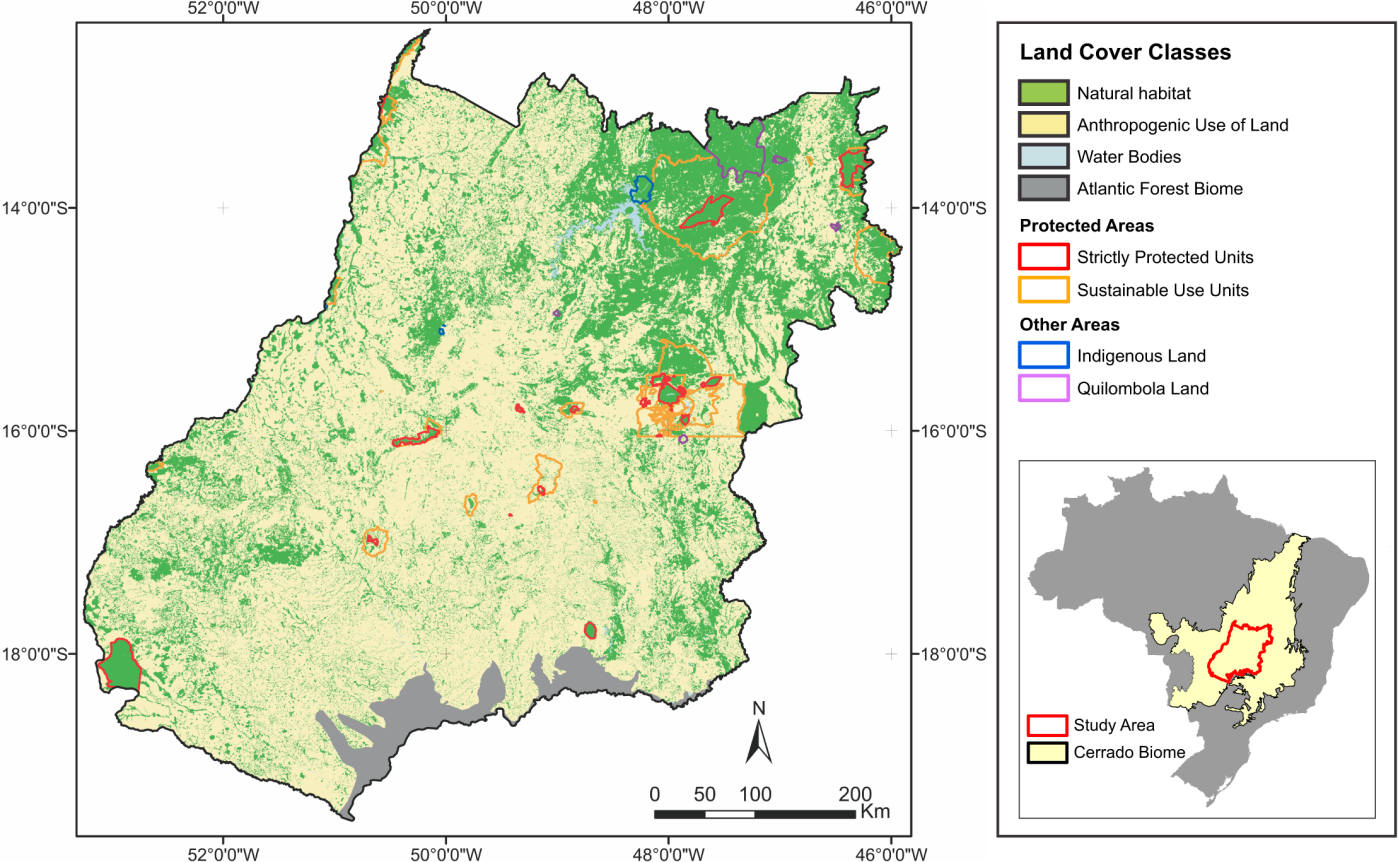
Protected areas (polygons) and land cover in the study region.

The area of interest is the nuclear region of the Cerrado biome, defined by the intersection between the borders of Goiás, the Federal District, and the limits of Cerrado as defined by the Map of Brazilian Biomes [25]. This region occupies 335,000 km^2^, which is approximately 16.5% of the biome’s total area, and 4% (2.03 million km^2^) of the Brazilian continental territory (8.51 million km^2^). The area covers two units of the Federation: the Federal District and the largest part of Goiás (97% of its territory is within the Cerrado biome). An area of about 10,500 km^2^, which is located in the south of Goiás, is situated in the Atlantic Forest Biome, and, as a result, is not considered in this study (Fig 1).

The nuclear region is a typical Cerrado region with high environmental diversity with respect to soil types, geology, geomorphology, and seasonal climate as well as a heterogeneous mosaic of vegetation that encompasses forest, savannah, and grassland vegetation types [37,39–41]. The study area encompasses four main natural landscape types: (1) well-drained plains and plateaus dominated by savannas, with semi-deciduous forests and grasslands as secondary vegetation types (most of the study area); (2) plains dominated by deciduous and semi-deciduous forests with dense savanna as the secondary vegetation type; (3) hilly terrain dominated by savanna with deciduous forest and grasslands as the secondary types; and (4) a small region in the northwest of the study area characterized by poorly drained lowlands dominated by seasonally flooding savannas [37]. Besides the landscape heterogeneity, the study area includes regions in eight of Cerrado’s 15 ecological units as defined by Silva, Fariñas, Felfili, & Klink (2006) [37] as well as three of the six floristic regions of Cerrado as defined by Ratter et al. (2003) [35].

The Cerrado core region was colonized in the 1930s, with the occupation of the south of Goiás and later in the 1950s, with the relocation of the Brazilian capital to Brasília [42,43]. The occupation of this region was accelerated and expanded after the 1950’s, increasing its population from 1.01 million people in 1950 to 8.57 million in 2010 [44]. The study area presents contrasts related to its occupation history in comparison with other sub-regions of the Cerrado, like the southeastern region, with occupancy from the eighteenth century, and northern and western regions of the Biome, with most recent occupation, from the 1980's [42,45].

The Brazilian government played a decisive role in this occupation process of the analyzed area by providing land, subsidies, and technical assistance; promoting the development of large cattle ranches and agriculturally used areas; and supported the installation of two main urban centers (Goiânia and Brasília) and an extensive transport network infrastructure. Approximately 66% of the study area, approximately 223,000 km^2^, belongs to anthropic land use classes, while the remaining 34% belong to natural habitat areas [24]. Most of LUCC events in the region occurred before the last decade. Between 2002 and 2010 around 3.18% were converted to anthropogenic use [24].

### Current Brazilian Law on Protected Areas

In Brazil, protected areas were defined through the National Protected Areas System (SNUC) [46] and divided in two main groups: Strictly Protected Areas and Sustainable Use Areas. These groups differ with respect to the level of restriction on the use of biodiversity components and the level of access to the area. Strictly Protected Areas vary between International Union for Conservation of Nature (IUCN) categories I to III, while Sustainable Use Areas vary between categories IV and VI [47,48] (S2 Table). The majority of protected areas are a part of federal, state, or municipal governmental spheres and differ depending on the agents responsible for the regulation and/or management of the unit. Most of the protected areas are in the federal or state sphere, while a mere few in the municipal governmental sphere were recognized and recorded by the National Register of Protected Areas (CNUC), the agency that is responsible for the registration of existing protected areas in Brazil. Some of the SNUC areas are owned by individuals (private natural heritage reserves, natural monuments, wildlife refuges) since the compatibility between the objectives of these protected areas and their use can be ensured and submitted for the supervision of government environment agencies. The units of the SNUC system have primary objectives of biological conservation and account for accomplishment of biodiversity targets of the UN Convention on Biological Diversity [49,50].

Since the National Protected Areas Plan (PNAP) was instituted by a presidential decree [51], other areas that do not belong to the SNUC system were included in the planning and implementation of a broad integrated biodiversity conservation and management strategy [52]. The plan highlights the importance of the Indigenous Lands and Quilombola Lands (occupied by traditional groups of African origin), which occupy extensive natural areas that are well maintained and managed in a diversified manner by human populations usually in a sustainable manner. The contributions of the Indigenous Lands to biological and habitat conservation has already been explored [12,20,38], while the importance of the Quilombola Lands remains virtually unevaluated. Sociocultural diversity, but not biological or habitat conservation, is the primary objective of both of these types of units. These units are also not committed to a special restriction for land use as they can be converted to anthropogenic use since the limits prescribed by the regular environmental law, in particular, the Brazil Forest Code, are respected [53]. Despite the fact that these types of areas are actually, or at least potentially, important for biodiversity conservation, these areas are not recognized by the CBD or IUNC system as protected areas. In this study, they are referred to as "Other areas”.

### Protected Areas and Other Areas Network

The protected areas in Goiás and in the Federal District account for about 12% of the protected areas in the Cerrado biome. In the study area, there are 56 units, with a total area of 22,966 km^2^ (6.85% of the study area) divided into groups, the Strictly Protected (1.11%) and Sustainable Use (6.33%) [54]. The spatial overlap between these categories of restriction accounts for 1,993 km^2^ (0.59% of the study area). In the study area, 15 federal units and 41 state units occupy a similar total area (S3 Table). RPPN’s or municipal protected areas were not included because no units are recorded with available spatial data in the official registering agency (CNUC).

The first protected area was created in 1949 in the study area, and in 2010, the total of protected areas reached 56 units (S1 Fig). The creation rate of protected areas was lower until the second half of the 1990s (0.6 protected areas per year) and increased substantially in the later period (2.5 protected areas per year, between 1998 and 2010). This pattern can also be observed, particularly for Sustainable Use areas, whose number increased from 11 units in 1998 to 35 units in 2010.

Among protected area units that met the requirements of the conducted sampling (S4 Table), as described in the “GIS Processing and Protected Area Sample” section, the protected area units age (yeas from its creation) ranged from 8 to 65 years, with a median of 16 years from creation (units created in 1998 included). In turn, the protected area units exhibited a size range from 4.86 km^2^ to 7,950.05 km^2^ with a positive skewed distribution (mean = 587.96, median = 166.74 km^2^, Kurtosis = 21.38, Skewness = 4.44). We considered the median value to define the size groups as described in the “Matching Analysis” section.

The SNUC protected areas share the study area with 4 units of Indigenous Land with an area of 412 km^2^ (0.12% of the study area) [55] and 5 units of Quilombola Lands with an area of approximately 2,750 km^2^ (0.82% of the study area). No spatial overlap between these areas or between Indigenous Lands or Quilombola Lands with protected areas occurred [56].

### Effect of Protected Areas on Habitat Protection

Evaluations of the effect of protected areas on prevention of natural habitat conversion occur via a wide variety of approaches, with studies evaluating different scales of analysis, covering different environments, and adopting different methodologies. The main factors distinguishing these analyses are: 1) the response variable or estimator considered in the analysis, 2) the definition of the counterfactual element, 3) use of controls of statistical bias, and 4) evaluation of the hidden bias according to other variables not considered in the study [57].

As for the response variable, this type of evaluation considers events as direct effects or correlated with anthropic changes in natural habitats. Most studies use estimators based on the calculation of areas of deforestation or based on the presence of classes of anthropic use (e.g., urban, agricultural, artificial pastures, and naked soil) and analyze the absolute data (converted areas) for the same period or between different periods. Rates of change between periods are also used as well as the probability of change between different states (anthropic vs. natural) in conjunction with other environmental characteristics (e.g., slope, distance to roads, and distance to towns) [58–60]. In addition to the calculation of the cleared area itself, these authors also use events correlated to changes in the land use, such as occurrence/frequency of fires [12,14,61].

Given the fact that protected areas are regions that receive special treatment, it is necessary to choose an element for comparison of the response variable in order to quantify the effects of the treatment. According to Ferraro et al. (2007) [62], the ideal comparison would be between the delimited protected areas; however, no unit of this kind is present in this region. As we have no direct access to this information, we need to properly define a based reference for measuring deforestation or avoiding anthropic conversion. Based on the classifications of Joppa & Pfaff (2010) [10] and Geldmann et al. (2013) [9], the counterfactual elements, or comparison elements, can be: “compared to nearby time”, “compared to everywhere”, “compared to nearby land” and “compared to similar habitats outside”.

Although comparison to nearby regions (buffer analysis) or comparison to the entire outside region may be used more frequently, the most promising strategy is one that considers regions that are environmentally and socially similar to those in the protected areas as counterfactual elements. The main advantage of this strategy is a better efficiency in the control of two main sources of bias: the spatial correlation bias and the autocorrelation bias [10,13,62,63].

The first bias stems from the non-randomness of the allocation of protected area units throughout the territory [64] and the influence of covariates on the anthropic conversion. The non-randomness of the allocation of these areas is related to a great number of units in regions that are less than adequate for human use [65–68], with less political opposition to instalment [69,70]. In addition, this bias results from the defining aspect of these areas, whose purpose, by definition, is to preserve regions with attributes of biological and environmental importance, which are not randomly distributed in the territory [71–73]. The control of social and environmental characteristics is thus necessary in order to not confuse the influence of protected areas with the influence of covariates on the probability of change in land use. Using a sampling model that fails to consider controls of this bias may weaken the results obtained on the effects of the protected areas, since these analyses would then compare regions with different environmental characteristics and with different social dynamics, resulting in different probabilities for anthropic conversion [10,13,62,64]. In general, the lack of control in these sources of bias results in the overestimation of the effectiveness of the protected areas, given that these areas are being compared with regions that usually have a higher probability of anthropic conversion than the treated samples.

The second bias that should be controlled stems from the fact that land use in a particular region may affect the probability of use and occupation in nearby regions, in a manner related to spatial autocorrelation [62]. In the case of protected areas, it has been noted that regulation and restriction of the land use by political agents has resulted in changes in the use of nearby regions that are not regulated [74,75]. The so-called “spillover effect” is a type of bias related to spatial autocorrelation [62,63]. In the context of the study of protected areas, the spillover effect refers to the wide shift of anthropic activities caused by public regulation of the land use [10,60]. Ferraro et al. (2007) classified the spillover effect as negative when protected area policies resulted in anthropic pressure on the surrounding area (e.g., an increase in economic, agricultural, or touristic activities) or as positive (e.g., creation of new protected areas nearby and/or prevention of the increase in the region’s transport infrastructure).

Comparison with regions directly influenced by protected area units results in statistical bias in favor of the event that is being assessed (e.g., the influence of protected areas on the estimator, the total cleared area). Sampling designs that do not seek to control this source of bias may result in an underestimation of the effect of those units based on the hypothesis that the protected areas reduce the probability of natural habitat conversion in nearby regions or an overestimation based on the hypothesis that protected areas promote an increase in natural habitat conversion in nearby regions.

The bias caused by these two sources can be reduced significantly by choosing a proper control group in a sampling design that mimics randomized experimental studies. In this kind of study, the evaluation of different treatments is made by the random designation of sampling units to the control group and to the treatment group, and the effects of the treatment group will be evaluated. These sampling units differ only in the characteristics that are important to the study, and other covariates are equally distributed between both groups. Observational studies, such as this, aim at replicating this type of experiment by obtaining a treated control group with similarly distributed covariates [76].

When the matching method is applied to observational studies [77,78], the control group is formed ex post. The general purpose of this method is to find, in a wide variety of sampling units that are not part of the treatment, those which share as many similarities as possible to units that received treatment and in particular, variables that are closely related to the relevant characteristics that exert an influence on the response variable [76,79]. In the context of this study, most similarities are defined as the “smaller distance” in terms of a multivariate measure, considering all the characteristics that individualize the land and that have an influence on the probability of anthropic conversion.

This approach allows for the reduction of the effects of covariates-induced statistical bias on the estimation of protected area effects on natural habitat conversion, as demonstrated by Andam et al. (2008) [57] and Ferraro et al. (2007) [62], This approach also avoids spatial autocorrelation bias, as the sampling units are located in a space that is not adjacent to the protected areas.

The matching method has four main aspects [76,79]:

1. the definition of covariates that influence the outcome of the response variable;
2. the definition of a proximity measure, used to define a good pair for the comparison. The Propensity Score or the Mahalanobis Score are commonly used;
3. implementation of a matching method (e.g., nearest neighbor, Kernel, or Ray);
4. evaluation of the quality of the results obtained from the matching.

**Fig 2:**
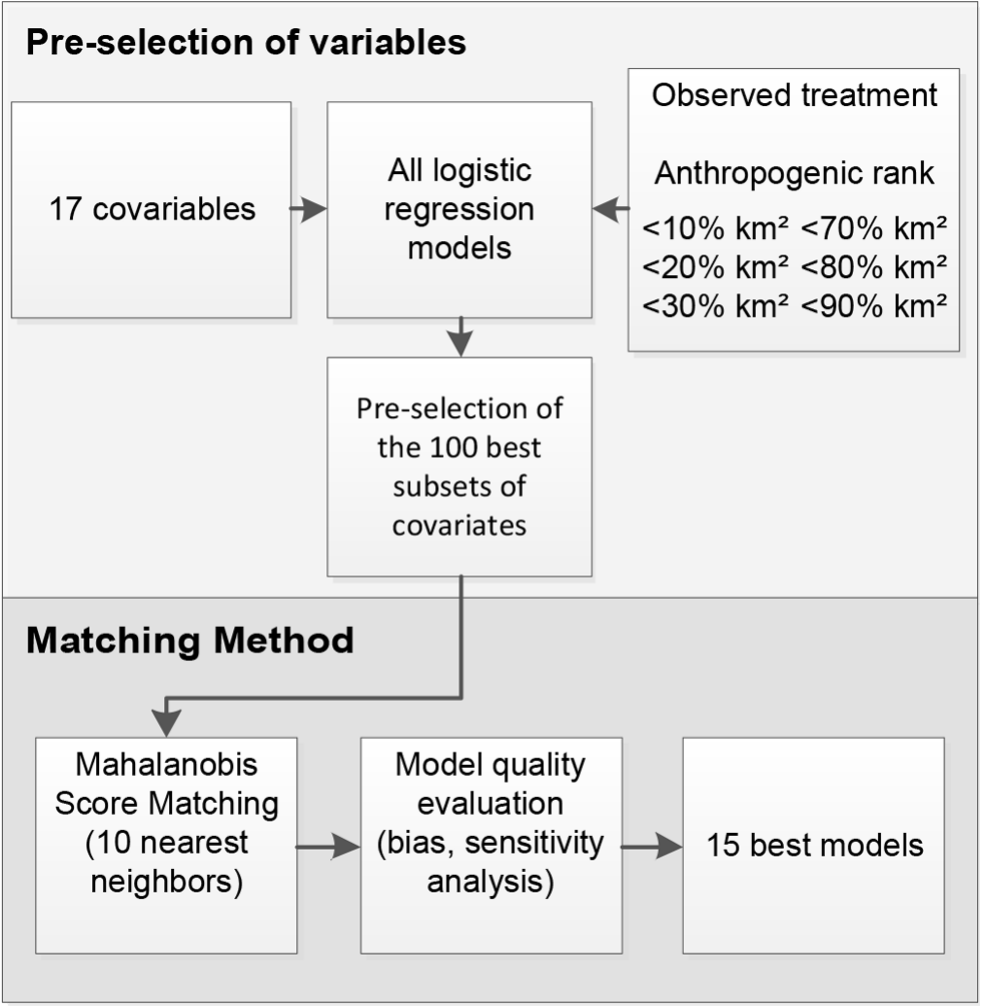
Flowchart of methodology used in the study. 1) Pre-selection of the best covariates groups. 2) Realization and evaluation of the matching quality.

Based on the characteristics of the adopted method of analysis as well as the theoretic and practical requirements relevant to the study theme, the methodology was divided into the following domains: definition and procurement of the variables of interest (treatment variable, response variable, and covariates), pre-selection of a group of covariates that better explain the variation in the conversion to anthropic use in the study region, and realization and evaluation of the matching quality and estimation of the results (Fig 2).

### GIS Processing and Sampling Strategy

We defined a square grid of 1000 m by 1000 m in order to obtain the information related to the considered covariates (S5 Table). The regular grid covered the entire study area, including the intersection between Goiás/Federal District and the limits of the Cerrado [25]. Each of the grid cells represents one of the sampling unit. For each grid unit, we obtained the eventual occurrence of protected areas (type, group, and government sphere), the anthropic use of the area in each cell, and 17 independent covariates (S5 Table).

**Fig 3:**
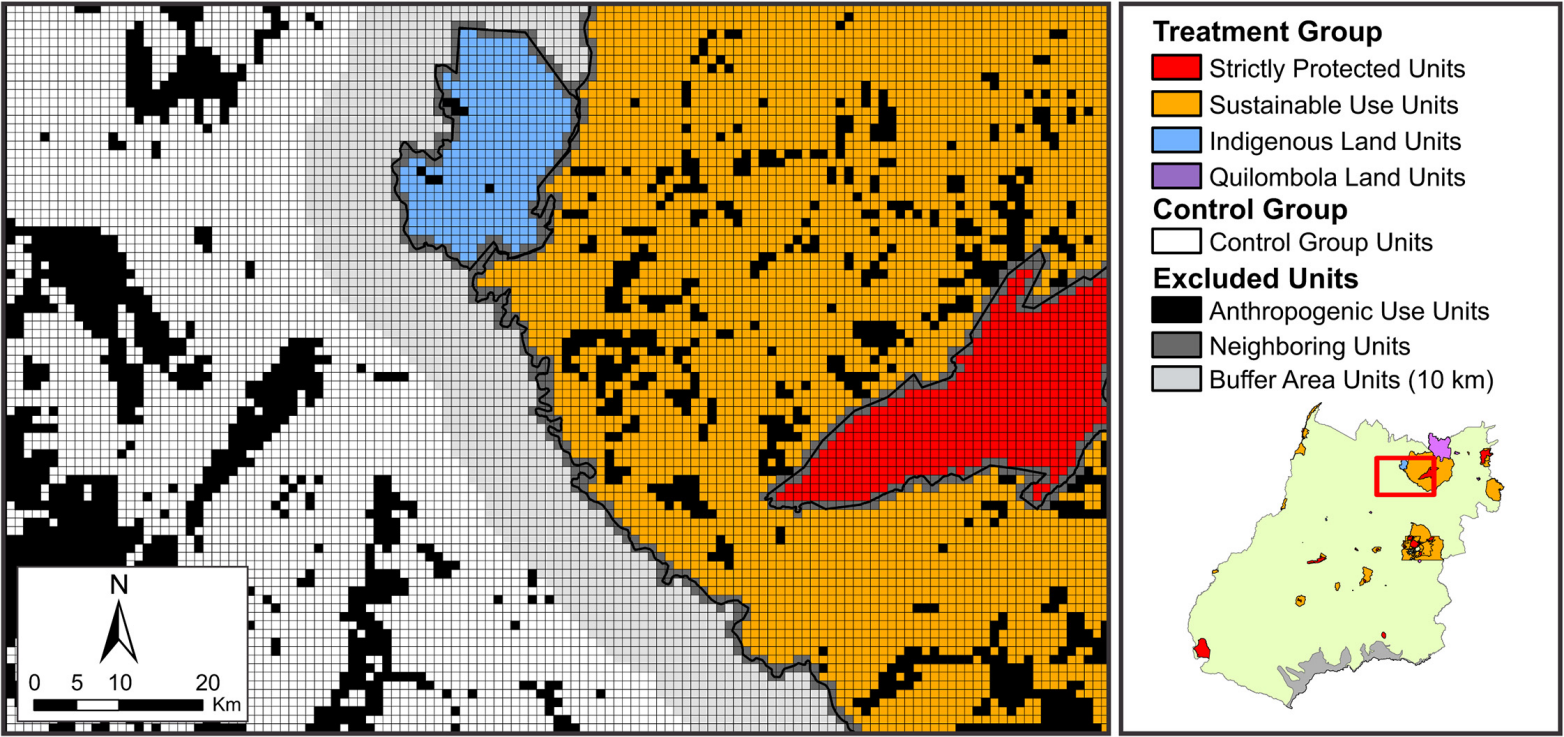
Regular grid with cells of 1000 m by 1000 m. The map depicts the sampling units in protected areas, the sampling units excluded from the analysis, and the sampling units from the 10-km buffer.

The map of protected areas was obtained from official spatial information (digital archives) and made available by governmental agencies. This region comprises Protected Areas [54], Indigenous Lands [55], and Quilombola Lands [56]. As for the case of spatial overlap between different protected areas, those with more restrictions were kept. Only the sampling units that were fully included within the borders of a protected area were considered, and therefore, cells in neighboring regions between protected areas and areas that were not protected or between protected areas of different types or groups were not considered (Fig 3).

The spatialization of covariates of interest resulted in a regular grid with 327,457 sampling units, of which 22,531 were units inside protected areas and 304,926 units were in the remaining territory. Of these, 39 units in protected areas, 2 units in Indigenous Lands, and 5 units in Quilombola Lands met the requirements of the conducted sampling (S4 Table). The selected units enabled the inclusion of at least one complete unit in the regular grid inside the protected area limits classified as “natural”.

46 LANDSAT TM and ETM + were processed and analyzed in order to gather land use types for the years 1986 (23 images acquired between 1985–1986) and 1996 (23 images acquired between 1995–1996). The units sampled, within the study area, were classified as "natural" when the entire cell coverage was presented with native vegetation, and "anthropic", when all or part of the cell showed conversion to anthropic use. For the years 2002 and 2008, the classification of sample units were obtained from classifications done previously by the National Program of Satellite Monitoring [24].

For the control group, only those units out of a 10 km buffer were considered, and only those sample units classified as "natural", both in the treatment group, as well in the control group, were subjected to the statistical analysis [80,81]. In addition, to control the autocorrelation and spillover effects, this study looked at only LUCC events occurring between dates on which there were defined cohorts and date references (2010). This way, any deforestation that may have occurred prior to the creation of the protected areas, and which could skew the data, was excluded.

The sample design resulted in the formation of four groups of protected areas: those with creation previous to 1986; those with creation between 1986 and 1996; those with creation between 1996 and 2002; and those with creation between 2002 and 2008. Each protected area was added to its respective control group according to the nearest year after the date of its creation, resulting in: 2,537 sample units in the treatment group and 113.563 in the control group, for the year 1986; 879 sample units in the treatment group and 97.946 in the control group, for the year 1996; 9.802 sample units in the treatment group and 61.054 in the control group, for the year 2002; 434 sample units in the treatment group and 52.460 in the control group, for the year 2008. There was a minimum of 6 units in the control group for each unit in the treatment group, the amount normally considered appropriate to carry out the matching process.

All GIS digital image processing was conducted using ARCGIS 10.1, and ENVI 4.7 software. The South America Albers Equal Area Conic projection, with SAD 69 as Datum, was adopted as the spatial referencing system.

### Independent Variables and Model Selection

According to Geist & Lambin (2002) [82] and Lambin et al. (2001) [83], natural habitat conversion is related to distal and proximal factors, locally and regionally, that originate when several characteristics in a social, political, economic, and cultural context are combined together and is influenced by environmental factors that prepare the environment for human action. Studies conducted in the Cerrado region revealed a relationship between anthropic conversion with agricultural expansion and the extension of urban and transport infrastructure, and this relationship was influenced by attributes in environment, demography, economy, and political institutions [39,42,45,84–86].

The present study considered variables that may influence anthropic conversion, and such variables included environment attributes (altitude, slope, classified slope, and distance to rivers), economic attributes (cattle raising production, grain production, local gross domestic product (GDP), GDP per capita, average rural income, and average urban income), socioeconomic attributes (total population, rural population, urban population, human development index (HDI), and institutional/infrastructure attributes (municipalities area, distance to towns, and distance to roads). Mentioned covariates were spatialized to the base of municipalities in the study area. The variables of total population, rural population, urban population, cattle raising production, and grain production were standardized by the municipalities area (S5 Table).

An ideal group of independent covariates define the characteristics of an environment to best determine the probability of anthropic conversion throughout the territory in the absence of a variable that would estimate the effect (the occurrence of protected areas). In order to select the best group of covariates, we submitted the 17 variables to a selection process based on 1) the combination of different variables in order to obtain all the possible sub-groups, 2) the performance of analysis using multiple logistic regression for different treatments, and 3) the evaluation of the results and the pre-selection of the 100 best groups of covariates to be matched subsequently.

For pre-selection of the 100 best subsets of covariates, we observed the average percentage of correct answers between the condition predicted by the model based on covariates and that actually observed in the territory, while also taking into account different degrees of anthropogenic impacts. Six different treatments were noted from the logistic regression models: 1) anthropic conversion of less than 10% of the sampling unit area, 2) anthropic conversion of less than 20% of the sampling unit area, 3) anthropic conversion of less than 30% of the sampling unit area, 4) anthropic conversion of more than 70% of the sampling unit area, 5) anthropic conversion of more than 80% of the sampling unit area, and 6) anthropic conversion of more than 90% of the sampling unit area. For each of these situations, the dependent variable Y for the sampling unit i received a value of 1 when the requirement was met and a value of 0 when the requirement was not met (e.g., for the treatment 2, sampling units with less than 20% of anthropic conversion was signed as 1 and more than 20% as 0). The percentage of agreement refers to the coincidence between the predicted condition (1 or 0) and the observed condition (1 or 0).

All analyses in the variables selection process were conducted using the R 3.0.2 software [87]. The 100 pre-selected groups were subsequently submitted to matching analysis.

### Matching Analysis

The estimation of Protected Area Effectiveness was based on the Mahalanobis score matching system, using the 10 nearest neighbors with a caliper of 0.25. Two distinct metrics were obtained; the average treatment effect on the treated (ATT), which presents absolute mean differences between the sampling units in the control group and the respective pairs of treatment groups; and the relative average effect (ATT%), the difference between the average conversion observed between the units of the control group and that observed for the respective pairs of the treatment group, divided by the average conversion of the control group. These two metrics are complementary. The first provides information on the magnitude of the differences between the two sample unit groups, while the second measures how far baseline conversion rates have been changed by the presence of PAs, allowing for the comparison of results for regions with different baselines.

The protected area units were subjected to analysis in different classes and subgroups with consideration for potentially relevant characteristics for effectiveness in avoiding anthropogenic conversion. These characteristics included unit type, protected area restriction groups, governmental sphere, time from establishment, and size. The defined subgroups were as follows: 1) protected area group (all units of SNUC) and other areas; 2) strictly protected units (SP) and sustainable use units (SU), 3) Federal sphere units (Fed) and state sphere units (Sta), 4) larger sized protected areas, units with a greater-than-median size (>size), and smaller sized protected areas, and units with less than the median size (<size), 5) PAs created before 1986 (<86), PAs created between 1986–1996 (<96), PAs created between 1996–2002 (<02), PAs created between 2002–2008 (<08). Each cohort, defined in the "GIS Processing and Sampling Strategy" section, was compared to the year 2010, and the results were grouped among the different categories using a weighted average, taken from the number of sampling units and the number of PAs.

For the selection of the 15 best covariate models that had the best characteristics in matching quality, we evaluated the bias, the ATT standard error, and the pseudo-R^2^ of the 100 pre-selected covariates groups as defined in the “Independent Variables and Models Selection” section. We analyzed these among the results obtained for each PA subgroup.

We addressed potential hidden biases through sensitivity analysis. The model that showed the best quality from the 15 selected models was submitted to analysis of the sensitivity of the results to hidden bias. The method proposed by Rosenbaum (2002) [88] explores the effect that the not-observed covariate u may have caused on the results for different levels of Rosenbaum’s sensitivity factor (Г). For any magnitude Г ≥ 1, the interval of possible p-values (Pmax and Pmin) and confidence interval of the treatment effect was calculated. The conclusions of the study are changed by the bias induced by covariates not observed for a given level of Г, for which the Pmin shows a reduced value and the Pmax shows an increased value (more than 0.05) [78]. Thus, in order to change the results of the study due to a covariate not considered, the bias was determined. Studies showed that an elevated Pmax (p>0.05), for levels of Г near 1 are considered too susceptible to the hidden bias, while higher levels of Г imply greater robustness of the results. For observational studies, a value of Г near 2 is considered within a scale from moderate to high.

For the evaluation of the occasional influence of the autocorrelation effect and spillover effect, similar to other “inside-outside” analyses [89–91], there was defined a 10km buffer from each protected area unit. These effects were accessed by comparing the protected area ATT and ATT% for two groups: the first with the exclusion from the analysis of control group sampling units inside the buffer (buffer group), and the second with inclusion of all sampling units in the analysis (non-buffer group). The difference between the ATT and the ATT%, between these groups was evaluated.

All statistical analyses related to the matching procedures and analysis of sensitivity to hidden bias were conducted with the Stata 12.0 software, using the “psmatch2” [92] and ‘Rbounds’[93] packages, respectively.

## Results

### Model Choice

The 100 best groups of covariates that were pre-selected via multiple logistic regressions exhibited a small difference in the percentage of anthropic classes that were correctly classified (Table 1). Even without any posterior adjustment, the logistic regression models had a high percentage of correct classifications for classes with a low level of anthropic conversion (<30%). In fact, as many as 86.31% of these classes were correctly classified, while the classes with a higher level of anthropic conversion (>70%) showed a lower percentage of correct classification, with only 67.70% of these were correctly classified.

**Table 1:** Percentage of different anthropic classes that were classified correctly for the 100 best groups of pre-selected data.

	Antropogenic Rank					
	<10%	<20%	<30%	>70%	>80%	>90%
Mean	86.09%	83.99%	81.23%	69.09%	65.78%	65.07%
Standard deviation	0.21%	0.20%	0.21%	0.42%	0.51%	0.70%
Highest	86.31%	84.24%	81.58%	69.60%	66.58%	66.05%
Lowest	85.19%	83.10%	80.41%	67.70%	64.08%	63.64%

After the groups of pre-selected covariates were matched and the quality attributes (bias, standard error, and pseudo R^2^) were evaluated, the 15 best models were chosen (S6 Table), and among these, the one with the best characteristics was identified. Each group of covariates among the 15 best selected models was found to contain between 4 and 8 different covariates. Covariates with similar information (e.g., the rural population and total population of the municipality) did not appear in the same group. In the entire group (all 15 models), 12 of the 17 covariates from this study were found. The covariates of altitude, municipality GDP, urban income, rural population and urban population did not occur on the 15 selected models. The group of covariates that showed the best relative performance for the matching’s quality characteristics were classified slope, population density, cattle production, grain production, distance to roads and distance to river.

The sub-group containing the 15 best models showed common support between treatment and control groups, with Mahalanobis values ranging from 3.23 (5 percentile) to 20.13 (95 percentile) with a higher frequency for the lower values. For estimating the ATT effect 15,122 out of 127,349 distinct available sampling units were effectively used as control groups. Most of the control sampling units were set at a distance of 100 km from the protected areas, although some occurred at greater distances (S2 Fig). When applying a 0.25 caliper, there was a maximum of 1,462 sample units belonging to the treatment group and excluded from matching (off support). This left 12,190 samples for evaluation (on support), when considering the more comprehensive subgroup ("Protected Areas"). The worst relation in percentage was observed for the subgroup "Smaller size", where 31% of the sample units from the treatment group did not find a pair in the control group that was within caliper limits. Even with the exclusion of such sampling units, no protected area, as a whole, was excluded from the analysis.

These models enabled a significant reduction in bias after the matching process, accounting for a reduction of at least 94% and up to 99% of the bias before the matching process. For the 15 best model groups, an average bias of 5.57 remained after matching, while the best model had a bias of 1.51 remaining. The pseudo R^2^ after the matching process also showed a significant reduction, while the pseudo R^2^ before the matching analysis had an average reduction of more than 85%, reaching values of less than 5% for most of the protected area types and groups. These factors indicate a good control group for achieving the effect of protected areas.

The results obtained for the best model showed little sensitivity to influence of covariates not analyzed in the study, and were even resistant to the influence of elevated bias (Г near 2) or highly elevated bias (Г near or greater than 3) caused by covariates not seen in the study (S7 Table). Minor performances were observed, in general, for the group formed between 1996 and 2002, in which most had lower levels of resistance to the hidden bias (Г between 1.8 and 2.0). Even these low performing groups were highly resistant to the influence of hidden bias. Another factor that highlights the robustness of the model is the low variation of ATT and ATT% effect between the best model and the group of 15 best models (S6 Table). Even with the inclusion or exclusion of some covariates, results remained similar.

**Fig 4:**
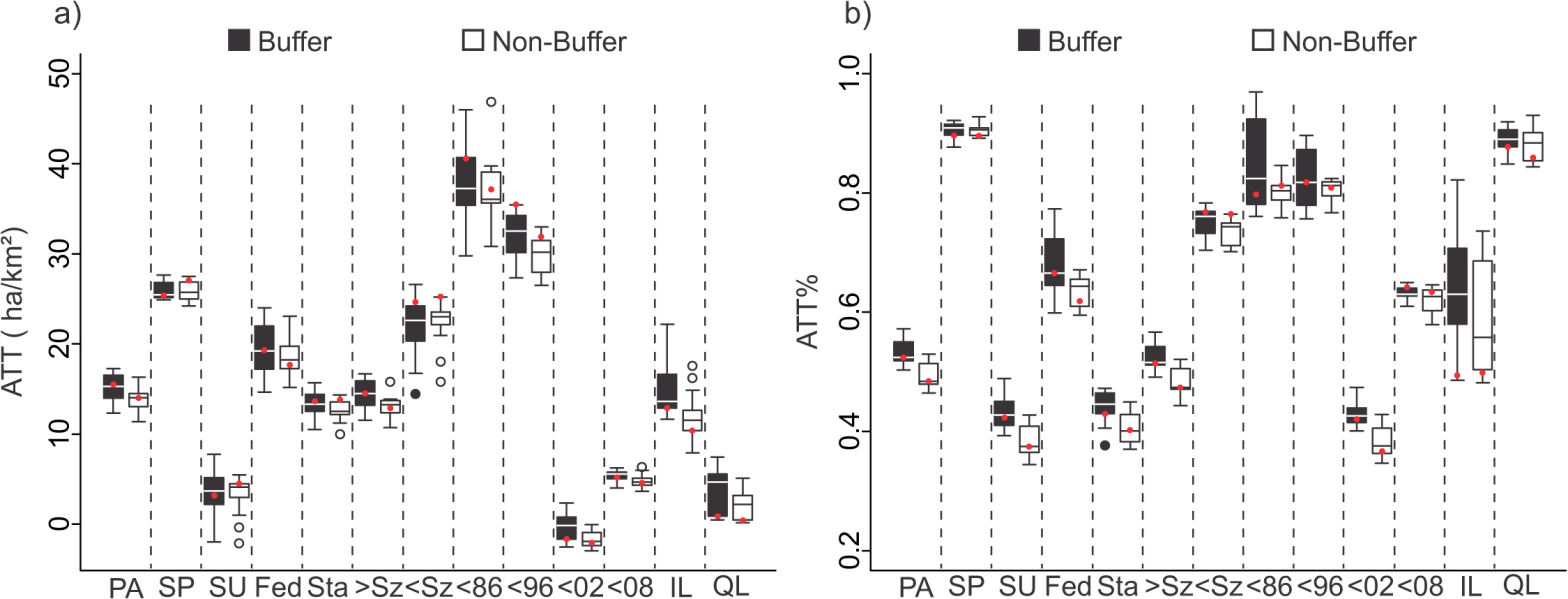
The ATT (a) and ATT% (b) for the 15 best models (box plots) and for the best model (red dots) of buffer and non-buffer subgroups. PA–Protected Area, SP–Strictly Protected Areas, SU–Sustainable Use Areas, Fed–Federal Units, Sta–State Units, >Sz–Larger size PAs, <Sz–Smaller size PAs, <86—Before 1986, <96—Between 1986–1996, <02—Between 1996–2002, <08—Between 2002–2008.

Differences observed between results obtained for buffer and non-buffer groups, point to a slight influence of autocorrelation effect, or spillover effect for some of the subgroups. A significant difference in ATT and ATT% was observed for the largest subgroup, the state's sphere subgroup, and the 1996–2002 cohort (Fig 4 and S8 Table), which showed significant negative differences between buffer and non-buffer groups. A significant negative difference suggests a higher probability for the region near these target units to be affected by conversion to anthropic classes, as opposed to similar areas that are not near the protected areas (the negative spillover / autocorrelation effect). In order to avoid any influence by autocorrelation or spillover effects, we considered only results that were outside the buffer region.

### Effectiveness of Protected Areas and Other Areas

The ATT and ATT% showed that protected areas have a positive effect on natural habitat preservation (Table 2). A negative difference of 15.49 ha/km^2^ (standard error = 0.79) was estimated in relation to the control group, suggesting that, in the absence of those areas, the study area would have an average increase of 15.49 hectares of anthropic land per km^2^. As for the relative effect, protected areas performed better than non-protected areas (ATT% = -0.55). When compared to Indigenous Lands and Quilombola lands, protected areas displayed differing results among observed indices (Table 2), with a higher effect for absolute index and less of an effect on relative index. The absolute index tends to overestimate the differences between groups when control units do not present similar LUCC levels, while the relative index does not show the same behavior in these cases. The lowest effectivity value, for the Quilombola Lands, on the absolute index is mainly due to the fact that these units are located in areas with more of a preserved natural habitat(average of anthropogenic controls for units of 8.3 ha/km^2^ for Quilombola Lands and 16.97 ha / km^2^ for Protected Areas; S9 Table). In the case of Indigenous Lands, slightly higher averages were found than those observed for protected areas for the control group (18.9 ha/km^2^), meaning that the absolute index (ATT) adequately reflects the difference in effectiveness between the groups.

**Table 2:** Average treatment effect for SNUC Protected Areas as estimated from the best data group.

Group/Subgroup	PA Units	S.U		After Matching					Before Matching			
		On	All	ATT	S.E.	ATT%	Bias	P. R^2^	Dif.	S.E.	Bias	P. R^2^
**Type Group**												
Protected Areas	39	12190	13652	-15.49	0.79	-0.55	1.38	0.01	-21.27	0.63	38.30	0.28
Indigenous Lands	3	316	316	-12.87	0.94	-0.88	2.70	0.31	-44.66	2.10	74.50	0.46
Quilombola Lands	5	2389	2389	-0.86	0.49	-0.63	1.70	0.13	-11.13	0.36	48.80	0.67
**Restriction Group**												
Strictly Protected	15	2534	2917	-25.34	1.67	-0.91	1.48	0.03	-29.69	1.83	43.32	0.21
Sustainable Use	24	9656	10735	-3.18	6.56	-0.45	1.03	0.06	-9.67	1.00	55.52	0.38
**Sphere Group**												
Federal Sphere	15	4292	5461	-19.28	0.82	-0.72	1.72	0.05	-22.26	0.79	45.46	0.29
State Sphere	24	7893	8191	-13.62	1.29	-0.45	1.52	0.01	-21.02	1.06	46.70	0.31
**Size Group**												
Larger Size	19	11839	13136	-14.49	0.86	-0.54	1.45	0.05	-20.57	0.67	41.30	0.31
Smaller Size	20	351	516	-24.65	3.47	-0.78	1.91	0.03	-20.48	2.83	55.05	0.24
**Age Group**												
Before 1986	10	1831	2537	-40.55	0.87	-0.97	1.20	0.02	-42.90	0.76	51.20	0.37
Between 1986–1996	8	688	879	-35.44	1.23	-0.87	0.30	0.01	-37.89	1.26	36.10	0.20
Between 1996–2002	15	9243	9802	1.68	0.52	-0.44	1.90	0.01	-9.01	0.27	36.70	0.32
Between 2002–2008	6	428	434	-5.16	1.02	-0.63	1.30	0.01	-4.59	0.84	24.80	0.14

ATT, Absolute Effect; ATT%, Relative Effect; S.E., Standard Error; P. R^2^, Pseudo R^2^; Dif., Unmatched Difference.

**Fig 5:**
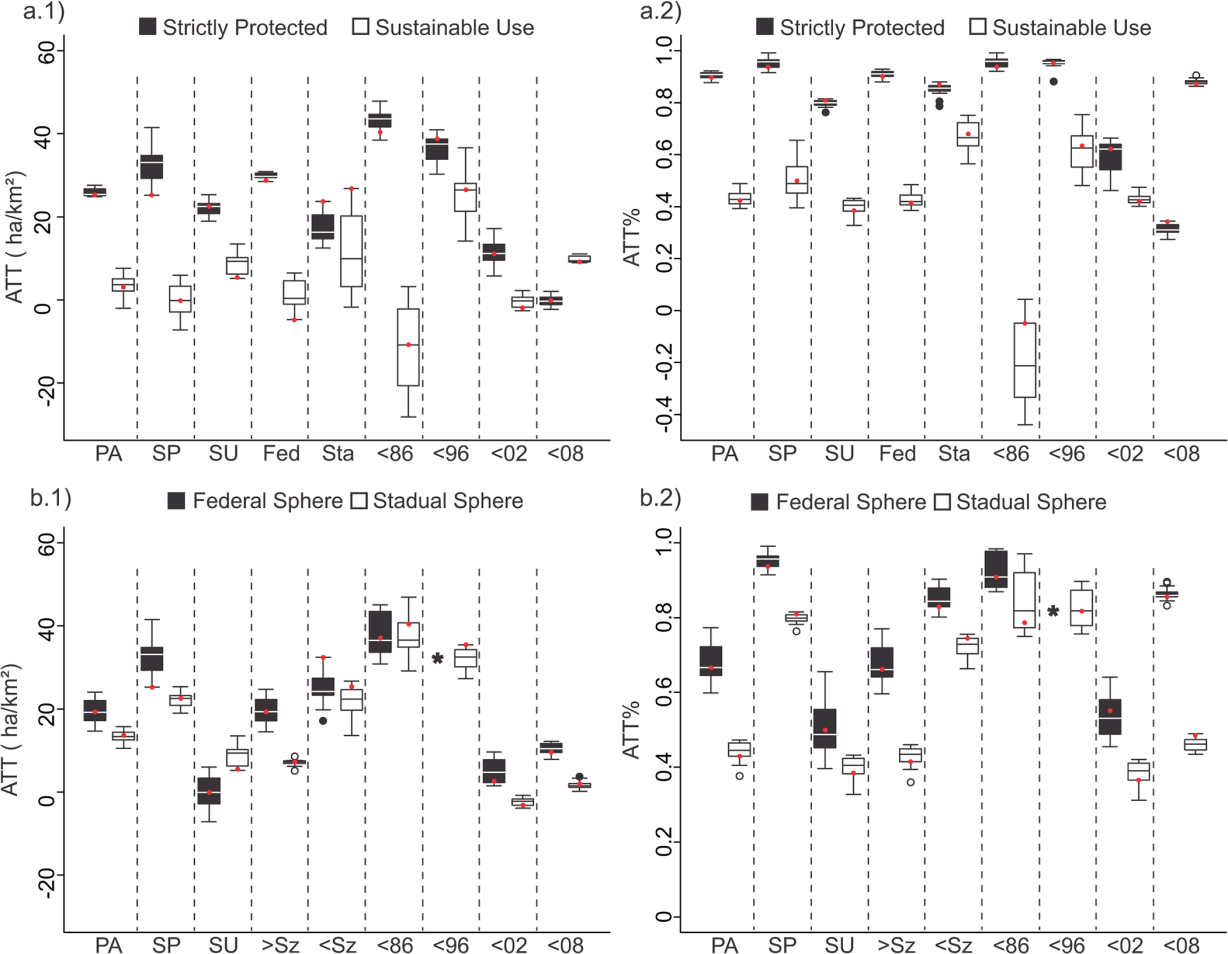
The absolute mean ATT and ATT% for the 15 best models (box plots) and for the best model (red dots) regarding restriction and government sphere groups. a) restriction group (a.1 –ATT, a.2 ATT%), b) government sphere group (b.1 –ATT, b.2 ATT%). PA–Protected Areas, SP–Strictly Protected Areas, SU–Sustainable Use Areas, Fed–Federal Units, Sta–State Units, >Sz–Larger sized PAs, <Sz–Smaller sized PAs, <86—Before 1986, <96—Between 1986–1996, <02—Between 1996–2002, <08—Between 2002–2008.

The effectiveness of the protected areas was markedly different with respect to restriction, governmental sphere, size and cohort groups, both in terms of absolute effect as well as the relative effect. In the restriction group, an important variation was observed between strictly protected units (ATT 25.34 ha/km^2^, standard error 1.67 ha/km^2^, ATT% -0.91) and sustainable use units (ATT 3.18 ha/km^2^, standard error 6.56 ha/km^2^, ATT% -0.45), demonstrating that the sustainable use groups were less effective. Despite the fact that the units in the control group, used to estimate the effectiveness of sustainable use units, present important differences compared to those for strictly protected units (average of 10.87 ha/km^2^ for sustainable use units and 40.65 ha/km^2^ to strictly protected units) there was a correlation between the general patterns obtained for both indices. These differences remained in the presence of observed influence of other characteristics, such as government sphere, size and most cohort subgroups (Fig 5 and S10 Table).

Similar results were observed for the groups related to governmental sphere. For these groups, greater effectiveness data was obtained for federally protected area units (ATT 19:28 ha/km^2^, standard error 0.82 ha/km^2^, ATT% -0.72) compared to state sphere units (ATT 13.62 ha/km^2^, standard error 1.29 ha/km^2^, ATT% -0.45). For both, absolute index and relative index, there was a difference of approximately 30% between the mentioned groups, even considering that the average for the control groups differed significantly (29.08 ha/km^2^ for federal sphere units and 10.72 ha/km^2^ is state sphere units) (Table 2 and S9 Table). Lower effectiveness of the state units was found repeatedly among most subgroups (restriction, size subgroups and cohorts), except for those created after 1996 (Fig 5 and S11 Table).

**Fig 6:**
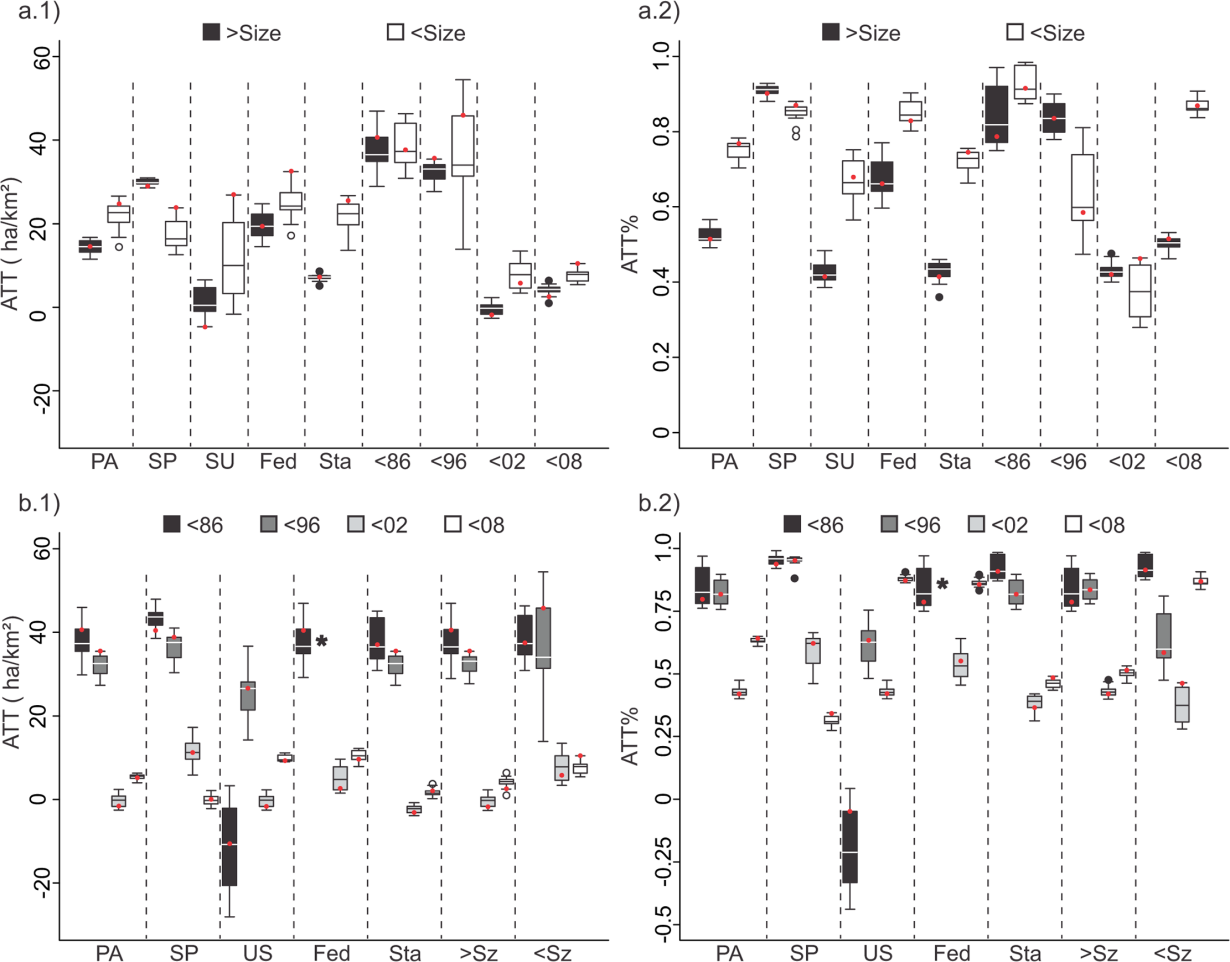
The absolute mean ATT and ATT% for the 15 best models (box plots) and for the best model (red dots) regarding size group and cohorts. a) size group (a.1 –ATT, a.2 ATT%), b) cohorts (b.1 –ATT, b.2 ATT%). PA–Protected Areas, SP–Strictly Protected Areas, SU–Sustainable Use Areas, Fed–Federal Units, Sta–State Units, >Sz–Larger sized PAs, <Sz–Smaller sized PAs, <86—Before 1986, <96—Between 1986–1996, <02—Between 1996–2002, <08—Between 2002–2008.

The overall size of the unit also affected the effectiveness of protected areas in the study region. Smaller areas (ATT 24.65 ha/km^2^, standard error 3:47 ha/km^2^, ATT% -0.78) generally showed better performance in protecting the natural habitat than larger areas (ATT 14:49 ha/km^2^, standard error 0.86 ha/km^2^, ATT% -0.54). For the strictly protected units subgroup, however, there was greater effectiveness with larger areas (ATT 29.83 ha/km^2^, standard error 1.66 ha/km^2^, ATT% -0.91) compared to those of smaller size (ATT 17.39 ha/km^2^, standard error 6:37 ha/km^2^, ATT% -0.85) (Fig 6 and S12 Table). Larger protected areas were found in regions with lower anthropogenic change, with 16.73 ha/km^2^ being the average LUCC for sample units of the control group, while those for the larger group had a mean value of 25.10 ha/km^2^. This difference had no significant impact on the used indices, which showed similar patterns for most observed subgroups (Fig 6, S9 Table and S12 Table).

Among the protected areas cohorts, the largest differences were observed between areas created before 1996 ("Before 1986" and "Between 1986–1996" cohorts) and those with a later creation date ("Between 1996–2002" and "Between 2002–2008" cohorts). In general, the older areas showed greater effectiveness than the units with more recent creation, a pattern that was repeated for both the absolute index and for the relative index. Older protected areas (created before 1996) avoided more than 35.00 ha / km^2^ of anthropogenic conversion (ATT% lower than -0.85), while protected areas, which were created more recently (after 1996), accounted for less than 10:00 ha/km^2^ of saved area (ATT% higher than -0.65). The general pattern observed was varied, particularly for the sustainable use subgroup. In this case, units with most recent creation had a higher effectiveness compared to older units (Fig 5 and S13 Table).

## Discussion

The avoidance of anthropogenic conversion is not the ultimate test for the objectives fulfillment of protected areas. Biological conservation can be significantly compromised even by factors not directly related to natural habitat preservation, such as hunting [94] and climate change [28], and we cannot overlook the direct, indirect, or synergistic effects [27,30] of natural coverage protection. Therefore, it is important to evaluate whether protected areas cause substantial avoidance of anthropogenic conversion.

The results of this study confirm the importance of protected areas for natural habitat preservation, although these effects vary between different subgroups. A variation in the restriction group was noticed, and this finding was consistent with other recent studies of the Cerrado biome [20] or other regions [9,14,38,89]. Strictly protected areas possess a greater ability to avoid such conversion (25.34 ha/km^2^), but these areas occupy a reduced total area (about 15% of all protect areas in the study area). Meanwhile, the sustainable use areas, which are more numerous and with a larger occupied area (about 85% of all protected areas in the study area) was less effective at preventing such conversion (3.18 ha/km^2^) when compared to regions with similar relevant characteristics.

These results suggest that habitat conservation polices should give higher priority to strictly protected areas by increasing the total size of these units. It also suggests that sustainable use areas may not be the most appropriate instrument for preserving natural habitats, specifically in the Cerrado regions with high anthropogenic pressure. Our findings advocate for the creation, and designation of these units, in conjunction with improved habitat conservation policies, since positive effectiveness was significant (about 45% better than unprotected areas with same characteristics). The establishment of this type of SNUC subcategory may be an option, especially in cases where it is not feasible or desirable to create strictly protected areas [95]. In addition, the results suggest that current reclassification of protected area units to lower restriction groups in Brazil [23] may result in increased anthropogenic conversion rates. Nevertheless, a greater sampling effort is necessary in order to determine whether the low levels of effectiveness for the sustainable use group may be restricted to specific SNUC or IUCN subcategories (e.g., environment protection area–IUCN V).

Besides a difference among restriction groups, a consistent pattern was observed in the government sphere group. Several factors may be related to the lower effectiveness of preventing anthropic conversion in state sphere units. For example, a greater influence of local political and economic power on regulatory measures for land, besides a lower availability of economic, infrastructural, and personal resources for the maintenance of state protected areas. The confirmation of such factors, however, leans towards further inquiry at the local scale.

Furthermore, age and size classifications may play an important role in anthropogenic conversion avoidance. As usually observed [57], protected areas with more time elapsed since their establishment were more effective than those with less time since creation. Although the present study reinforces this assertion as a general rule, it was observed that certain types of units may respond differently to the elapsed time, such as the group of units for sustainable use, which presented lower effectivities in units created in a later period. In turn, as a general pattern, the smaller protected areas were more effective than the larger ones. Some groups, however, responded in the opposite way, as in the case of strictly protected areas. The result for the Cerrado core region is in opposition to findings obtained by Naughton-Treves et al. (2005) [3]. Those investigators reported a non-significant correlation of the protected area size with deforestation avoidance. Although, the higher effectiveness of smaller sized protected areas for some groups (e.g., stadual sphere units) cannot be understood in an isolated context without considering the greater importance of larger areas for biological conservation. The observed patterns regarding age and size groups may be influenced by management effectiveness and protected area resource availability [96].

The results obtained for indigenous lands in central Cerrado confirm, at a sub-regional level, those presented by Carranza et al. (2013) [20] for the entire biome. Although Indigenous Lands are not the type of unit that is commonly intended for environmental conservation, the obtained data suggests similar or higher effectiveness levels for them, as observed for the protected group areas. These findings agree with the results from other regions, including the Amazon biome, where this type of unit has been shown to yield effectiveness as high as that for the strictly protected groups [12]. As for the Quilombola Lands, a lower level of effectiveness was observed when compared with the Indigenous Lands. This observed result is mainly due to the location of this type of unit and the lack of representation of these lands in the study area. The Quilombola Lands investigated were concentrated in regions with low anthropogenic influence (north of the study area), which is reflected primarily in the absolute index. It is advisable to employ a larger sampling effort in order to understand the role played by this type of protected area in relation to the maintenance of natural habitat.

The matching method and the procedure used for choosing the best groups of covariates enabled a highly significant reduction of bias in the estimates of the protected area effectiveness toward anthropic conversion and ultimately reduced the influence of covariates. According to the sensitivity tests conducted, the results proved to be fairly resistant to the characteristics that were not considered in the study and have a potential influence on the probability of natural habitat conversion. In addition, the attainment of results for different sets of variables, and the implementation test for the selection of covariates, rather than the previous election of a single set of variables, contributes significantly to the stronger results in relation to hidden bias. This strategy also allowed for the observation of any casual variations in the results, when considering other characteristics of the region under analysis. Considering the rising availability of spatial information, the use of this methodology in the evaluation of the effects of protected areas on avoidance of anthropic conversion proves to be increasingly promising, enabling the achievement of more accurate results.

## Conclusions

The analyses in this study indicate that protected areas play an important role in protecting the overall natural habitat in the core region of the Cerrado biome. This despite the observance of a wide variation in the effectiveness between different protected area groups, protected areas and non-protected areas, and specially protected regions (Indigenous Lands and Quilombola Lands). Even units that do not have environmental conservation as their primary objective (Indigenous Lands) achieved high results in protecting the natural habitat. Among protected area groups, differences in effectiveness toward avoidance of anthropic conversion were associated with the degree of restriction in place and available access, government sphere, size and age of the protected areas. In line with the expectation that there is a difference between some of the groups (e.g., strictly protected and sustainable use), the estimated magnitude suggests that environmental policies that aim to reduce anthropogenic conversion of the natural habitat should focus mainly on strictly protected areas, as is the case in other biomes. Furthermore, these results also highlight a need for public policies intended to achieve greater integration and balance in management quality and resource availability between government spheres.

## Supporting Information

S1 Fig The establishment of Protected Areas on the study region between 1949 (first PA) and 2010. (DOCX)

S2 Fig Spatial distribution of values obtained for the Mahalanobis Distance Score in treated (inside PAs polygons) and not treated samplings. (DOCX)

S1 Table Biodiversity in the Cerrado biome: the recorded richness for plants, mammals, birds, reptiles, amphibians, and fish biological groups. (DOCX)

S2 Table Brazilian National System for Protected Areas (SNUC) and the International Union for Conservation of Nature (IUCN) categories of protected areas. (DOCX)

S3 Table Protected areas in the study region compared to the whole Cerrado biome. (DOCX)

S4 Table Protected areas, Indigenous Lands, and Quilombola Lands that met the requirements of the conducted sampling. (DOCX)

S5 Table Source and description of variables used in model selection and matching analysis. (DOCX)

S6 Table Results obtained for the 15 best models. (DOCX)

S7 Table Values of Pmax for levels of Г for the best data group (sensitivity test). (DOCX)

S8 Table Results for the non-buffer and buffer groups. (DOCX)

S9 Table Statistics for the sample units in the control group effectively used to obtain the effectiveness of protected areas. (DOCX)

S10 Table Results for the restriction groups with respect to government sphere, age, and size subgroups. (DOCX)

S11 Table Results for the government sphere groups with respect to restriction, age, and size subgroups. (DOCX)

S12 Table Results for the size groups with respect to restriction, government sphere, and age subgroups. (DOCX)

S13 Table Results for the cohorts with respect to restriction, government sphere, and size subgroups. (DOCX)
