# Supplementary figures and images for: The Role of Protected Areas in the Avoidance of Anthropogenic Conversion in a High Pressure Region: A Matching Method Analysis in the Core Region of the Brazilian Cerrado
Source: PLoS One. 2015 Jul 29;10(7):e0132582. doi: 10.1371/journal.pone.0132582 (PMC4519267; doi:10.1371/journal.pone.0132582)

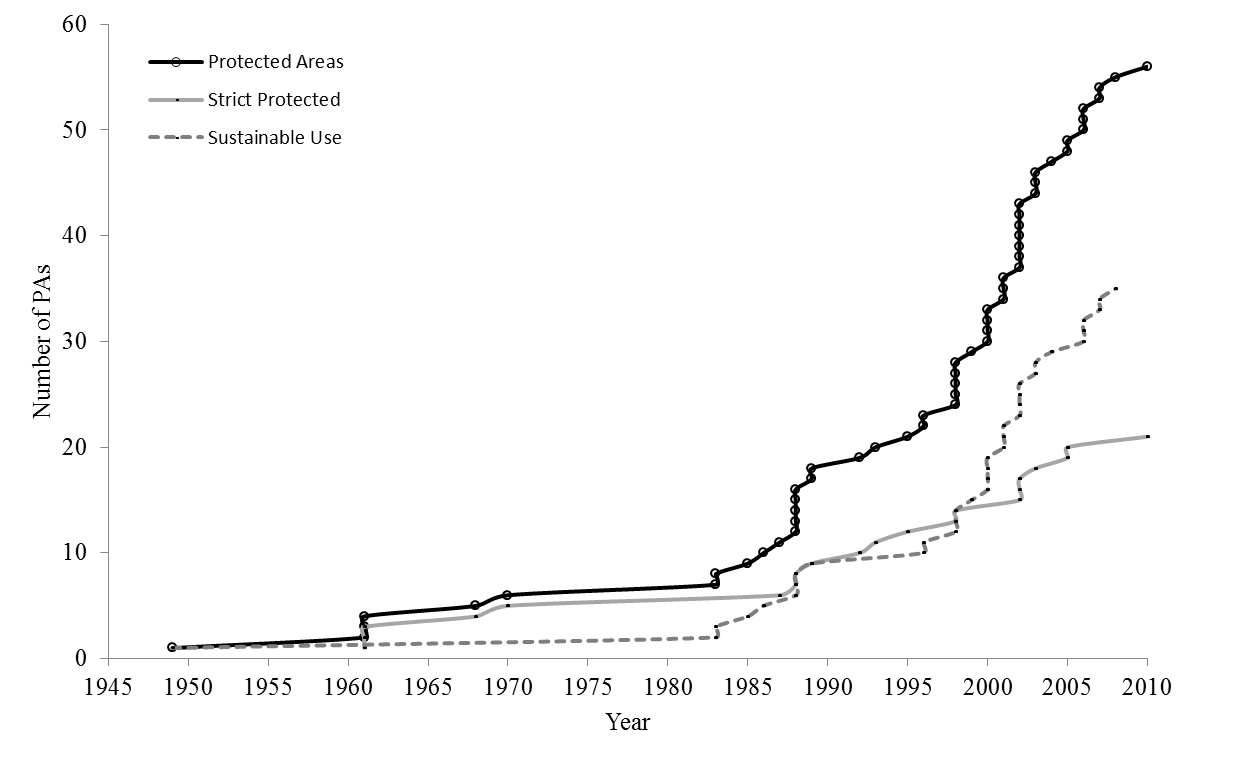


**Figure S1 –** The establishment of Protected Areas on the study region between 1949 (first PA) and 2010.

Supplement: S1 Fig — (DOCX) [file pone.0132582.s001.docx]
