# Supplementary material for: The Role of Protected Areas in the Avoidance of Anthropogenic Conversion in a High Pressure Region: A Matching Method Analysis in the Core Region of the Brazilian Cerrado
Source: PLoS One. 2015 Jul 29;10(7):e0132582. doi: 10.1371/journal.pone.0132582 (PMC4519267; doi:10.1371/journal.pone.0132582)
Supplement: S2 Fig — (DOCX) [file pone.0132582.s002.docx]

**
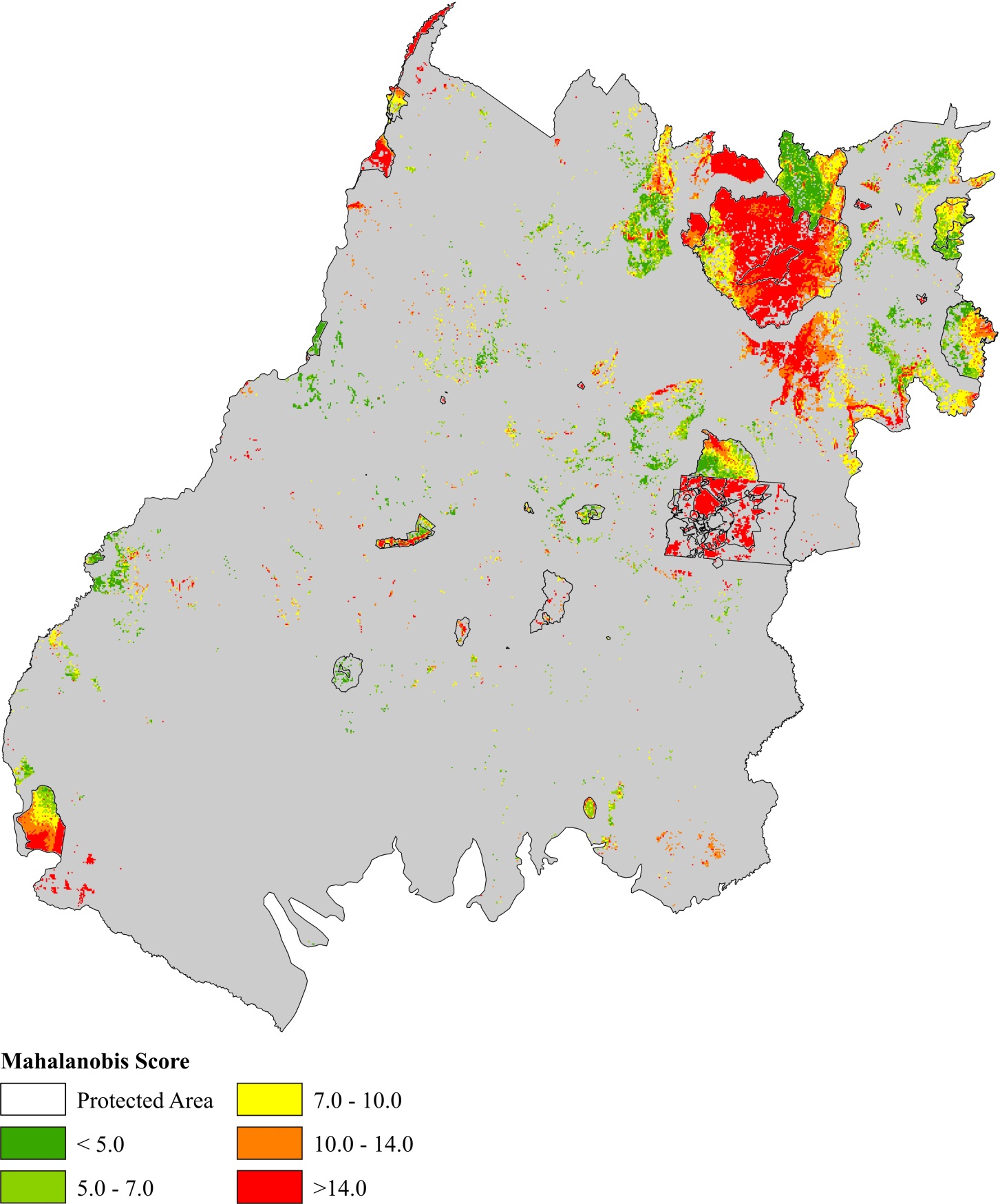
**

**Figure S2 –** Spatial distribution of values obtained for the Mahalanobis Distance Score in treated (inside PAs polygons) and not treated samplings.
