# Supplementary material for: The Role of Protected Areas in the Avoidance of Anthropogenic Conversion in a High Pressure Region: A Matching Method Analysis in the Core Region of the Brazilian Cerrado
Source: PLoS One. 2015 Jul 29;10(7):e0132582. doi: 10.1371/journal.pone.0132582 (PMC4519267; doi:10.1371/journal.pone.0132582)
Supplement: S1 Table — (DOCX) [file pone.0132582.s003.docx]

**Table S1 –** Biodiversity in the Cerrado Biome: the recorded richness for plants, mammals, birds, reptiles, amphibians, and fish biological groups.

| Grups | Cerrado | Brazil | World^(11)^ | Cerrado/Brazil (%) | Cerrado/World(%) |
| --- | --- | --- | --- | --- | --- |
| Plants | 12,356^(1)^ | 43,535^(2)^ | 281,052 | 28.38 | 4.40 |
| Mammals | 251^(3)^ | 701^(3)^ | 5,501 | 35.81 | 4.56 |
| Birds | 864^(4)^ | 1,832^(5)^ | 10,064 | 47.16 | 8.59 |
| Reptiles | 247^(6)^ | 744^(7)^ | 9,789 | 33.20 | 2.52 |
| Amphibians | 152^(6)^ | 946^(8)^ | 7,044 | 16.07 | 2.16 |
| Fish | 1,200^(9)*^ | 3,598^(10)^ | 32,500 | 33.35 | 3.69 |
| Total | 15,070 | 51,356 | 345,950 | 29.33 | 4.35 |

**Source:** (1) Medonça et al. (2008); (2) (IJBRJ, 2013); (3) Paglia et al. (2012); (4) J. M. C. da Silva & Santos (2005);(5) CBRO (2011); (6) Brites, Faria, Mesquita, & Colli (2009); (7) Bérnils & Costa (2012); (8) Segalla et al. (2012); (9) Aguiar, Machado, & Marinho-Filho (2004);(10) Rosa & Lima (2008);(11) IUCN (2013).

* Estimated.

1. Medonça, R. C., Felfili, J. M., Walter, B. M. T., Silva Junior, M. C., Filgueiras, T. S., Nogueira, P. E., & Fagg, C. W. (2008). Flora vascular do bioma Cerrado: checklist com 12.356 espécies. In A. Scariot, J. C. Sousa-Silva, & J. M. Felfili (Eds.), Cerrado: Ecologia e Flora. (p. V.2). Brasília, DF: Embrapa Informação Tecnológica.
2. IJBRJ, J. B. do R. de J. (2013). Lista de Espécies da Flora do Brasil 2013. Retrieved October 28, 2013, from http://floradobrasil.jbrj.gov.br/
3. Paglia, A. P., Fonseca, G. A. B., Rylands, A. B., Herrmann, G., Aguiar, L. M. S., Chiarello, A. G., Patton, J. L. (2012). Lista Anotada dos Mamíferos do Brasil. Occasional Papers in Conservation Biology. Arlington, VA: Conservation International.
4. Silva, J. M. C. da, & Santos, M. P. D. (2005). A importância relativa dos processos biogeográficos na formação da avifauna do Cerrado e de outros biomas brasileiros. In A. Scariot, J. C. Sousa-Silva, & J. M. Felfili (Eds.), Cerrado: Ecologia, Biodiversidade e Conservação. (pp. 218–233). Brasília, DF: MMA.
5. CBRO, C. B. de R. O. (2011). Lista das aves do Brasil. CBRO. Retrieved October 29, 2013, from http://www.cbro.org.br
6. Brites, V. L. de C., Faria, R. G., Mesquita, D. O., & Colli, G. R. (2009). The Herpetofauna of the Neotropical Savannas. In Unesco-Eolss (Ed.), Encyclopedia of Life Support Systems (Vol. X, p. Vol. X). Paris: Unesco-Eolss.
7. Bérnils, R. S., & Costa, H. C. (2012). Brazilian reptiles: list of species. Version 2012.2. Sociedade Brasileira de Herpetologia. Retrieved June 03, 2013, from http://www.sbherpetologia.org.br
8. Segalla, M. V., Caramaschi, U., Cruz, C. A. G., Garcia, P. C. A., Grant, T., Haddad, C. F. B., & Langone, J. (2012). Brazilian amphibians - List of species. Sociedade Brasileira de Herpetologia. Retrieved October 28, 2013, from www.sbherpetologia.org.br
9. Aguiar, L. M. de S., Machado, R. B., & Marinho-Filho, J. (2004). A Diversidade Biológica do Cerrado. In L. M. de S. Aguiar & A. J. A. de Camargo (Eds.), Cerrado: ecologia e caracterização. (pp. 19–42). Brasília: Embrapa Cerrados.
10. Rosa, R. S., & Lima, F. C. T. (2008). Os peixes brasileiros ameaçados de extinção. In A. B. M. Machado, G. M. Drummond, & A. P. Paglia (Eds.), Livro vermelho da fauna brasileira ameaçada de extinção. (pp. 9–285). Brasília, DF: MMA.
11. IUCN. (2013). IUCN Red List of Threatened Species: Summary Statistics for Globally Threatened Species. Retrieved October 28, 2013, from http://www.iucnredlist.org/about/summary-statistics
