# Supplementary material for: The Role of Protected Areas in the Avoidance of Anthropogenic Conversion in a High Pressure Region: A Matching Method Analysis in the Core Region of the Brazilian Cerrado
Source: PLoS One. 2015 Jul 29;10(7):e0132582. doi: 10.1371/journal.pone.0132582 (PMC4519267; doi:10.1371/journal.pone.0132582)
Supplement: S2 Table — (DOCX) [file pone.0132582.s004.docx]

**Table S2 -** Brazilian National System for Protected Areas (SNUC) and the International Union for Conservation of Nature (IUCN) categories of protected areas.

| **SNUC Group** | **SNUC Category** | **Acronym** | **IUCN Category** |
| --- | --- | --- | --- |
| Strictly Protected Areas | Ecological Station | ESEC | Ia |
|  | Biological Reserve | REBIO | Ia |
|  | National Park | PARNA | II |
|  | Natural Monument | MN | III |
|  | Wildlife Refuge | REVIS | III |
| Sustainable Use Areas | Area of Relevant Ecological Interest | ARIE | IV |
|  | Private Natural Heritage Reserve | RPPN | IV |
|  | Environmental Protection Area | APA | V |
|  | Sustainable Development Reserve | RDS | VI |
|  | Fauna Reserve | REFAU | VI |
|  | Extractive Reserve | RESEX | VI |
|  | National Forest | FLONA | VI |

**Source:** MMA. (2007). Informe Nacional sobre Áreas Protegidas no Brasil. (MMA, Ed.).
