# Supplementary material for: The Role of Protected Areas in the Avoidance of Anthropogenic Conversion in a High Pressure Region: A Matching Method Analysis in the Core Region of the Brazilian Cerrado
Source: PLoS One. 2015 Jul 29;10(7):e0132582. doi: 10.1371/journal.pone.0132582 (PMC4519267; doi:10.1371/journal.pone.0132582)
Supplement: S3 Table — (DOCX) [file pone.0132582.s005.docx]

**Table S3 –** Protected areas in the study region compared to the whole Cerrado biome.

| **Group** | **Study Area ^(1)^** | | | **Cerrado ^(2)*^** | | |
| --- | --- | --- | --- | --- | --- | --- |
|  | **Num.** | **Area (km²)** | **%** | **Num.** | **Area (km²)** | **%** |
| Federal Strictly Protected | 4 | 2,400 | 0.72% | 22 | 41,166 | 2.02% |
| State Strictly Protected | 17 | 1,319 | 0.39% | 86 | 16,943 | 0.83% |
| **Strictly Protected** | **21** | **3,719** | **1.11%** | **119** | **62,798** | **3.08%** |
| Federal Sustainable Use | 11 | 9,210 | 2.75% | 27 | 17,683 | 0.87% |
| State Sustainable Use | 24 | 12,030 | 3.59% | 76 | 90,117 | 4.43% |
| **Sustainable Use*** | **35** | **21,240** | **6.33%** | **95** | **109,921** | **5.40%** |
| Reference Area (km²) | - | 335,364 | - | - | 2,036,448 | - |
| Overlapping Area | - | 1,993 | 0.59% | - | 3,222 | 0.16% |
| SNUC Protected Area | 56 | 22,966 | 6.85% | 214 | 169,497 | 8.32% |

Source: (1) Obtained from the processing of spatial information provided by CNUC (MMA, 2013); (2) CNUC/MMA (2013).

* RPPN’s units excluded.
