# Supplementary material for: The Role of Protected Areas in the Avoidance of Anthropogenic Conversion in a High Pressure Region: A Matching Method Analysis in the Core Region of the Brazilian Cerrado
Source: PLoS One. 2015 Jul 29;10(7):e0132582. doi: 10.1371/journal.pone.0132582 (PMC4519267; doi:10.1371/journal.pone.0132582)
Supplement: S4 Table — (DOCX) [file pone.0132582.s006.docx]

**Table S4 –** Protected areas, Indigenous Lands, and Quilombola Lands that met the requirements of the conducted sampling.

| **ID** | **Name** | **Type** | **Group** | **Government Sphere** | **IUCN Category** | **Year of creation** | ***Area (km²)** |
| --- | --- | --- | --- | --- | --- | --- | --- |
| 1 | Reserva Biológica Da Contagem | SNUC PA | Strictly Protected | Federal | Ia | 2002 | 34.49 |
| 2 | Parque Nacional Da Chapada Dos Veadeiros | SNUC PA | Strictly Protected | Federal | II | 1961 | 647.96 |
| 3 | Parque Nacional Das Emas | SNUC PA | Strictly Protected | Federal | II | 1961 | 1295.93 |
| 4 | Parque Nacional De Brasília | SNUC PA | Strictly Protected | Federal | II | 1961 | 422.54 |
| 5 | Estação Ecológica De Águas Emendadas | SNUC PA | Strictly Protected | State | Ia | 1968 | 95.77 |
| 6 | Estação Ecológica Do Jardim Botânico | SNUC PA | Strictly Protected | State | Ia | 1992 | 45.03 |
| 7 | Parque Estadual Altamiro De Moura Pacheco | SNUC PA | Strictly Protected | State | II | 1993 | 31.39 |
| 8 | Parque Estadual Da Serra De Caldas Novas | SNUC PA | Strictly Protected | State | II | 1970 | 121.59 |
| 9 | Parque Estadual Da Serra Do Jaraguá | SNUC PA | Strictly Protected | State | II | 1998 | 28.29 |
| 10 | Parque Estadual Da Serra Dourada | SNUC PA | Strictly Protected | State | II | 2003 | 286.43 |
| 11 | Parque Estadual De Paraúna | SNUC PA | Strictly Protected | State | II | 2002 | 33.35 |
| 12 | Parque Estadual De Terra Ronca | SNUC PA | Strictly Protected | State | II | 1989 | 569.83 |
| 13 | Parque Estadual Do Araguaia | SNUC PA | Strictly Protected | State | II | 2002 | 45.51 |
| 14 | Parque Estadual Do Descoberto | SNUC PA | Strictly Protected | State | II | 2005 | 19.33 |
| 15 | Parque Estadual Dos Pirineus | SNUC PA | Strictly Protected | State | II | 1987 | 28.38 |
| 16 | Área De Relevante Interesse Ecológica Capetinga/Taquara | SNUC PA | Sustainable Use | Federal | IV | 1985 | 20.57 |
| 17 | Área De Proteção Ambiental Da Bacia Do Rio Descoberto | SNUC PA | Sustainable Use | Federal | V | 1983 | 303.90 |
| 18 | Área De Proteção Ambiental Da Bacia Do Rio São Bartolomeu | SNUC PA | Sustainable Use | Federal | V | 1983 | 809.80 |
| 19 | Área De Proteção Ambiental Das Nascentes Do Rio Vermelho | SNUC PA | Sustainable Use | Federal | V | 2001 | 1743.43 |
| 20 | Área De Proteção Ambiental Do Planalto Central | SNUC PA | Sustainable Use | Federal | V | 2002 | 4228.63 |
| 21 | Área De Proteção Ambiental Meandros Do Araguaia | SNUC PA | Sustainable Use | Federal | V | 1998 | 744.34 |
| 22 | Floresta Nacional Da Mata Grande | SNUC PA | Sustainable Use | Federal | VI | 2003 | 20.10 |
| 23 | Floresta Nacional De Brasília | SNUC PA | Sustainable Use | Federal | VI | 1999 | 93.36 |
| 24 | Floresta Nacional De Silvânia | SNUC PA | Sustainable Use | Federal | VI | 1949 | 4.86 |
| 25 | Reserva Extrativista Do Recanto Das Araras De Terra Ronca | SNUC PA | Sustainable Use | Federal | VI | 2006 | 119.82 |
| 26 | Reserva Extrativista Lago Do Cedro | SNUC PA | Sustainable Use | Federal | VI | 2006 | 173.37 |
| 27 | Arie Parque Jk | SNUC PA | Sustainable Use | State | IV | 1996 | 21.79 |
| 28 | Apa Da Bacia Dos Ribeirıes Do Gama E Cabeça De Veado | SNUC PA | Sustainable Use | State | V | 1986 | 166.75 |
| 29 | Apa De Cafuringa | SNUC PA | Sustainable Use | State | V | 1988 | 309.04 |
| 30 | Apa Do Lago Paranoá | SNUC PA | Sustainable Use | State | V | 1989 | 157.37 |
| 31 | Área De Proteção Ambiental Da Serra Das Galés E Da Portaria | SNUC PA | Sustainable Use | State | V | 2002 | 462.80 |
| 32 | Área De Proteção Ambiental Da Serra Dourada | SNUC PA | Sustainable Use | State | V | 1998 | 153.80 |
| 33 | Área De Proteção Ambiental Do Encantado | SNUC PA | Sustainable Use | State | V | 2003 | 94.44 |
| 34 | Área De Proteção Ambiental Dos Pireneus | SNUC PA | Sustainable Use | State | V | 2000 | 191.83 |
| 35 | Área De Proteção Ambiental João Leite | SNUC PA | Sustainable Use | State | V | 2002 | 738.67 |
| 36 | Área De Proteção Ambiental Pouso Alto | SNUC PA | Sustainable Use | State | V | 2001 | 7950.05 |
| 37 | Área De Proteção Ambiental Serra Da Jibóia | SNUC PA | Sustainable Use | State | V | 2000 | 171.62 |
| 38 | Área De Proteção Ambiental Serra Geral De Goiás | SNUC PA | Sustainable Use | State | V | 1996 | 320.60 |
| 39 | Floresta Estadual Do Araguaia | SNUC PA | Sustainable Use | State | VI | 2002 | 223.86 |
| 40 | Avá-Canoeiro | Indigenous Land | - | - | - | 1996 | 387.02 |
| 41 | Carretão I | Indigenous Land | - | - | - | 1990 | 16.80 |
| 42 | Baco Pari | Quilombola Land | - | - | - | 2006 | 31.48 |
| 43 | Família Magalhaes | Quilombola Land | - | - | - | 2004 | 54.90 |
| 44 | Kalungas | Quilombola Land | - | - | - | 2005 | 2613.43 |
| 45 | Mesquita | Quilombola Land | - | - | - | 2006 | 42.86 |
| 46 | Tomas Cardoso | Quilombola Land | - | - | - | 2008 | 18.03 |

* Non-spatial overlapping area.
