# Supplementary material for: The Role of Protected Areas in the Avoidance of Anthropogenic Conversion in a High Pressure Region: A Matching Method Analysis in the Core Region of the Brazilian Cerrado
Source: PLoS One. 2015 Jul 29;10(7):e0132582. doi: 10.1371/journal.pone.0132582 (PMC4519267; doi:10.1371/journal.pone.0132582)
Supplement: S5 Table — (DOCX) [file pone.0132582.s007.docx]

**Table S5 –** Source and description of variables used in model selection and matching analysis.

| **Variable** | **Acronym** | **Variable type** | **Units** | **Source** | **Year** | **Geographic scale** | **Description** | **Data access** |
| --- | --- | --- | --- | --- | --- | --- | --- | --- |
| Antropogenic area | - | Response | km² | PMDBBS /IBAMA | 2010 | 1:250000 | Deforestation maps from the satellite Brazilian Biome Deforestation Monitoring Project – IBAMA. | siscom.ibama.gov.br/monitorabiomas/cerrado/index.htm |
| Protected Area | PA | Treatment | binary | CNUC/MMA | 2008 | 1:250000 | Spatial information and registration data of conservation units. | http://mapas.mma.gov.br/i3geo/datadownload.htm |
| Indigenous Lands | IL | Treatment | binary | FUNAI | 2008 | 1:250000 | Spatial information and registration data of Indigenous Lands. | http://mapas.funai.gov.br/ |
| Quilombola Lands | QL | Treatment | binary | INCRA | 2008 | 1:250000 | Spatial information and registration data of Quilombola Lands | http://acervofundiario.incra.gov.br |
| Altitude | alt | Covariable | m | SIEG | 2005 | 1:250000 | Processed information from contour lines, elevation points, hydrography, water bodies and boundaries of Goiás State. | http://www.sieg.go.gov.br/ |
| Slope | slp | Covariable | percentage | SIEG | 2005 | 1:250000 | Processed information from contour lines, elevation points, hydrography, water bodies and boundaries of Goiás State. | http://www.sieg.go.gov.br/ |
| Classified slope | slp_clas | Covariable | slope classes | SIEG | 2005 | 1:250000 | Processed information from contour lines, elevation points, hydrography, water bodies and boundaries of Goiás State. | http://www.sieg.go.gov.br/ |
| Distance to towns | d_town | Covariable | m | SIEG | 2009 | 1:250000 | Processed information from spatial data of cities. | http://www.sieg.go.gov.br/ |
| Distance to roads | d_road | Covariable | m | SIEG | 2009 | 1:250000 | Processed information from spatial data of roads. | http://www.sieg.go.gov.br/ |
| Distance to rivers | d_river | Covariable | m | SIEG | 2009 | 1:250000 | Processed information from spatial data of rivers. | http://www.sieg.go.gov.br/ |
| Municipality area | area | Covariable | km² | IBGE | 2010 | 1:250000 | Spatial information and registration data for municipalities. | http://www.ibge.gov.br/webcart/tabelas.php |
| Cattle production | cattle | Covariable | cattle units/km² | SIEG | 2010 | - | Registration data on the number of cattle. | http://www.sieg.go.gov.br/ |
| Grain production | grain | Covariable | kg/km² | SIEG | 2010 | - | Registration data on the grain production. | http://www.sieg.go.gov.br/ |
| Municipality GDP | gdp | Covariable | R$ | SIEG | 2010 | - | Registration data on the gross domestic product (GDP). | http://www.sieg.go.gov.br/ |
| GDP per capita | gdp_p | Covariable | R$ | SIEG | 2010 | - | Registration data on the GDP per capita. | http://www.sieg.go.gov.br/ |
| Rural income | inc_rural | Covariable | R$ | IBGE | 2010 | - | Registration data on the average rural income. | http://www.ibge.gov.br/webcart/tabelas.php |
| Urban income | inc_urb | Covariable | R$ | IBGE | 2010 | - | Registration data on the average urban income. | http://www.ibge.gov.br/webcart/tabelas.php |
| Total population | pop_t | Covariable | inhabitant/km² | IBGE | 2010 | - | Registration data on the total municipality population. | http://www.ibge.gov.br/webcart/tabelas.php |
| Rural population | pop_rural | Covariable | inhabitant/km² | IBGE | 2010 | - | Registration data on the rural population. | http://www.ibge.gov.br/webcart/tabelas.php |
| Urban population | pop_urb | Covariable | inhabitant/km² | IBGE | 2010 | - | Registration data on the urban population. | http://www.ibge.gov.br/webcart/tabelas.php |
| HDI | hdi | Covariable | HDI Index | IBGE | 2010 | - | Registration data on the total Human Development Index (HDI). | http://www.ibge.gov.br/webcart/tabelas.php |

GDP - Gross Domestic Product; HDI - Human Development Index; PMDBBS – Project of Deforestation Monitoring of Brazilian Biomes; IBAMA – Brazilian Institute of Environment and Renewable Natural Resources; CNUC - National Register of Protected Areas; MMA – Ministry of the Environment; FUNAI – National Indian Foundation; INCRA – National Institute of Colonization and Agrarian Reform; SIEG – Goiás State System of Geoinformation; IBGE – Brazilian Institute of Geography and Statistics.
