# Supplementary material for: The Role of Protected Areas in the Avoidance of Anthropogenic Conversion in a High Pressure Region: A Matching Method Analysis in the Core Region of the Brazilian Cerrado
Source: PLoS One. 2015 Jul 29;10(7):e0132582. doi: 10.1371/journal.pone.0132582 (PMC4519267; doi:10.1371/journal.pone.0132582)
Supplement: S6 Table — (DOCX) [file pone.0132582.s008.docx]

**Table S6 –** Results obtained for the 15 best models (10 km buffer excluded).

| **REF** | **Variables** | **Num. Var.** | **Multiple Logistic Regression** | | | | | | **Mahalanobis Matching** | | | | |
| --- | --- | --- | --- | --- | --- | --- | --- | --- | --- | --- | --- | --- | --- |
|  |  |  | **Correct classification/Anthropogenic Rank** | | | | | | **Protected Areas** | | | | |
|  |  |  | **<10%** | **<20%** | **<30%** | **>70%** | **>80%** | **>90%** | **ATT** | **S.E.** | **ATT%** | **Bias** | **P. R²** |
| 1 | slp_clas d_road pop_t cattle grain_d d_river | 6 | 85.93% | 83.84% | 81.04% | 69.25% | 66.13% | 65.85% | -15.49 | 0.79 | -0.55 | 1.38 | 0.01 |
| 2 | slp_clas d_town cattle grain_d d_river | 5 | 85.93% | 83.95% | 81.27% | 69.05% | 65.87% | 65.77% | -14.64 | 0.93 | -0.52 | 2.48 | 0.02 |
| 3 | slp_clas d_town d_road cattle grain_d d_river | 6 | 85.92% | 83.90% | 81.17% | 69.37% | 66.23% | 65.81% | -15.28 | 0.87 | -0.51 | 3.39 | 0.03 |
| 4 | slp_clas d_road cattle grain_d | 4 | 85.93% | 83.79% | 80.92% | 68.94% | 65.49% | 64.41% | -12.48 | 0.99 | -0.50 | 1.32 | 0.01 |
| 5 | slp_clas d_road cattle grain_d d_river | 5 | 85.94% | 83.86% | 81.04% | 69.15% | 66.14% | 65.73% | -12.33 | 0.79 | -0.52 | 1.64 | 0.01 |
| 6 | slp d_town d_road cattle grain_d d_river | 6 | 86.03% | 83.94% | 81.27% | 69.26% | 66.07% | 65.72% | -15.30 | 0.84 | -0.55 | 5.56 | 0.03 |
| 7 | slp_clas d_road cattle grain_d inc_rural d_river | 6 | 86.14% | 83.89% | 81.03% | 69.31% | 66.25% | 65.70% | -13.97 | 1.11 | -0.52 | 4.81 | 0.04 |
| 8 | slp_clas d_town d_road cattle grain_d inc_rural d_river | 7 | 86.14% | 84.00% | 81.31% | 69.54% | 66.34% | 65.80% | -14.89 | 1.09 | -0.54 | 4.41 | 0.06 |
| 9 | slp d_town d_road cattle grain_d inc_rural d_river | 7 | 86.24% | 84.14% | 81.46% | 69.49% | 66.22% | 65.72% | -14.78 | 1.07 | -0.53 | 7.72 | 0.07 |
| 10 | slp_clas d_town d_road cattle grain_d hdi d_river | 7 | 86.18% | 84.00% | 81.13% | 69.47% | 66.26% | 65.81% | -17.11 | 1.08 | -0.53 | 5.11 | 0.03 |
| 11 | slp_clas area d_road cattle grain_d d_river | 6 | 85.97% | 83.87% | 81.04% | 69.26% | 66.22% | 65.87% | -16.08 | 0.83 | -0.52 | 4.32 | 0.04 |
| 12 | slp_clas area d_town d_road cattle grain_d inc_rural d_river | 8 | 86.15% | 83.98% | 81.32% | 69.59% | 66.52% | 65.84% | -17.10 | 1.14 | -0.52 | 4.21 | 0.04 |
| 13 | slp cattle grain_d d_river | 4 | 86.00% | 83.91% | 81.23% | 68.60% | 65.67% | 65.33% | -12.32 | 0.79 | -0.51 | 2.06 | 0.01 |
| 14 | slp d_town d_road cattle grain_d hdi d_river | 7 | 86.24% | 84.08% | 81.30% | 69.32% | 66.09% | 65.75% | -17.25 | 1.05 | -0.57 | 8.33 | 0.03 |
| 15 | slp_clas d_town cattle gdp_p | 4 | 85.22% | 83.19% | 80.47% | 67.85% | 64.13% | 63.72% | -16.54 | 1.41 | -0.56 | 5.66 | 0.05 |

Municipality area (area), cattle production (cattle), classified slope (slp_clas), slope (slp), distance to roads (d_road), distance to rivers (d_river), distance to towns (d_town), grain production (grain), total population (pop_t), GDP per capita (gdp_p), HDI Index (hdi), rural income (inc_rural)

* ATT – Absolute Effect, ATT% - Relative Effect; S.E. – Standard Error; P. R² - Pseudo R².

**Table S7 –** (continuation)

| **REF** | **Mahalanobis Matching** | | | | | | | | | | | | | | | | | | | |
| --- | --- | --- | --- | --- | --- | --- | --- | --- | --- | --- | --- | --- | --- | --- | --- | --- | --- | --- | --- | --- |
|  | **Strictly Protected** | | | | | **Sustainable Use** | | | | | **Federal Sphere** | | | | | **State Sphere** | | | | |
|  | **ATT** | **S.E.** | **ATT%** | **Bias** | **P. R²** | **ATT** | **S.E.** | **ATT%** | **Bias** | **P. R²** | **ATT** | **S.E.** | **ATT%** | **Bias** | **P. R²** | **ATT** | **S.E.** | **ATT%** | **Bias** | **P. R²** |
| 1 | -25.34 | 1.67 | -0.91 | 1.48 | 0.03 | -3.18 | 6.56 | -0.45 | 1.03 | 0.06 | -19.28 | 0.82 | -0.72 | 1.72 | 0.05 | -13.62 | 1.29 | -0.45 | 1.52 | 0.01 |
| 2 | -25.44 | 1.75 | -0.88 | 5.33 | 0.03 | -4.52 | 1.36 | -0.42 | 2.90 | 0.23 | -18.87 | 1.05 | -0.65 | 1.31 | 0.02 | -13.69 | 1.26 | -0.44 | 4.95 | 0.09 |
| 3 | -27.07 | 1.77 | -0.90 | 3.14 | 0.03 | -5.16 | 1.21 | -0.40 | 3.64 | 0.23 | -19.42 | 1.02 | -0.65 | 3.59 | 0.04 | -14.54 | 1.16 | -0.42 | 5.03 | 0.08 |
| 4 | -25.25 | 1.91 | -0.90 | 1.94 | 0.01 | -0.48 | 1.37 | -0.39 | 3.38 | 0.21 | -14.63 | 0.99 | -0.60 | 1.48 | 0.02 | -12.55 | 1.46 | -0.43 | 2.20 | 0.09 |
| 5 | -25.15 | 1.53 | -0.92 | 2.70 | 0.01 | 0.32 | 1.11 | -0.41 | 1.80 | 0.21 | -15.52 | 0.81 | -0.62 | 1.59 | 0.02 | -11.73 | 1.13 | -0.46 | 1.61 | 0.04 |
| 6 | -27.54 | 1.73 | -0.92 | 5.76 | 0.04 | -5.25 | 1.14 | -0.45 | 5.41 | 0.23 | -19.21 | 1.01 | -0.68 | 4.84 | 0.05 | -14.51 | 1.10 | -0.47 | 6.84 | 0.08 |
| 7 | -26.21 | 1.87 | -0.91 | 3.02 | 0.04 | -2.30 | 2.21 | -0.42 | 13.52 | 0.23 | -17.21 | 1.19 | -0.66 | 5.65 | 0.05 | -12.94 | 1.53 | -0.45 | 6.54 | 0.11 |
| 8 | -25.59 | 2.02 | -0.91 | 4.76 | 0.08 | -3.69 | 2.04 | -0.44 | 11.32 | 0.26 | -18.63 | 1.33 | -0.67 | 4.63 | 0.07 | -13.59 | 1.44 | -0.46 | 8.61 | 0.14 |
| 9 | -25.92 | 2.01 | -0.91 | 5.73 | 0.10 | -3.51 | 1.86 | -0.43 | 13.20 | 0.26 | -18.32 | 1.33 | -0.65 | 4.22 | 0.07 | -13.33 | 1.38 | -0.46 | 9.15 | 0.15 |
| 10 | -25.08 | 2.23 | -0.88 | 5.86 | 0.08 | -2.19 | 5.17 | -0.44 | 4.04 | 0.22 | -23.61 | 1.32 | -0.76 | 5.76 | 0.05 | -12.89 | 1.38 | -0.41 | 4.96 | 0.08 |
| 11 | -27.67 | 1.65 | -0.90 | 3.94 | 0.02 | -6.52 | 1.18 | -0.42 | 9.28 | 0.25 | -19.44 | 0.83 | -0.67 | 4.99 | 0.10 | -15.71 | 1.22 | -0.43 | 5.82 | 0.09 |
| 12 | -26.80 | 2.33 | -0.88 | 6.26 | 0.09 | -4.34 | 5.26 | -0.43 | 3.93 | 0.25 | -23.84 | 1.40 | -0.76 | 7.30 | 0.11 | -12.87 | 1.45 | -0.38 | 3.07 | 0.10 |
| 13 | -24.88 | 1.55 | -0.91 | 2.35 | 0.01 | 1.95 | 1.11 | -0.40 | 4.36 | 0.20 | -16.27 | 0.83 | -0.61 | 2.13 | 0.02 | -10.50 | 1.11 | -0.44 | 2.59 | 0.04 |
| 14 | -25.16 | 2.21 | -0.90 | 7.58 | 0.09 | -4.22 | 5.14 | -0.49 | 7.20 | 0.28 | -24.03 | 1.33 | -0.77 | 7.73 | 0.04 | -12.32 | 1.31 | -0.47 | 7.96 | 0.09 |
| 15 | -25.02 | 2.47 | -0.92 | 4.53 | 0.05 | -7.72 | 2.15 | -0.47 | 12.38 | 0.25 | -21.97 | 1.69 | -0.70 | 4.97 | 0.08 | -14.41 | 1.78 | -0.47 | 7.00 | 0.12 |

ATT – Absolute Effect, ATT% - Relative Effect; S.E. – Standard Error; P. R² - Pseudo R².

**Table S7 –** (continuation)

| **REF** | **Mahalanobis Matching** | | | | | | | | | | | | | | | | | | | |
| --- | --- | --- | --- | --- | --- | --- | --- | --- | --- | --- | --- | --- | --- | --- | --- | --- | --- | --- | --- | --- |
|  | **Larger Size** | | | | | **Smaller Size** | | | | | **Before 1986** | | | | | **Between 1986-1996** | | | | |
|  | **ATT** | **S.E.** | **ATT%** | **Bias** | **P. R²** | **ATT** | **S.E.** | **ATT%** | **Bias** | **P. R²** | **ATT** | **S.E.** | **ATT%** | **Bias** | **P. R²** | **ATT** | **S.E.** | **ATT%** | **Bias** | **P. R²** |
| 1 | -14.49 | 0.86 | -0.54 | 1.45 | 0.05 | -24.65 | 3.47 | -0.78 | 1.91 | 0.03 | -40.55 | 0.87 | -0.97 | 1.20 | 0.02 | -35.44 | 1.23 | -0.87 | 0.30 | 0.01 |
| 2 | -13.87 | 0.99 | -0.51 | 1.89 | 0.03 | -22.62 | 3.05 | -0.73 | 6.33 | 0.14 | -35.41 | 1.38 | -0.78 | 1.60 | 0.03 | -29.89 | 1.22 | -0.78 | 5.20 | 0.03 |
| 3 | -14.58 | 0.92 | -0.50 | 3.54 | 0.04 | -23.70 | 2.99 | -0.76 | 6.33 | 0.12 | -37.09 | 1.42 | -0.80 | 5.30 | 0.04 | -32.54 | 1.05 | -0.80 | 3.30 | 0.04 |
| 4 | -11.93 | 1.05 | -0.49 | 1.31 | 0.03 | -17.64 | 3.11 | -0.72 | 3.95 | 0.15 | -30.12 | 1.17 | -0.76 | 2.50 | 0.03 | -30.56 | 1.67 | -0.78 | 1.30 | 0.03 |
| 5 | -11.75 | 0.84 | -0.51 | 1.69 | 0.03 | -16.72 | 2.72 | -0.71 | 1.08 | 0.09 | -29.80 | 0.90 | -0.78 | 2.30 | 0.03 | -30.13 | 1.17 | -0.78 | 1.00 | 0.03 |
| 6 | -14.62 | 0.89 | -0.54 | 5.65 | 0.04 | -23.64 | 2.91 | -0.76 | 7.13 | 0.11 | -37.11 | 1.37 | -0.82 | 7.60 | 0.05 | -33.38 | 1.02 | -0.80 | 4.70 | 0.04 |
| 7 | -13.21 | 1.11 | -0.52 | 4.32 | 0.05 | -20.33 | 3.61 | -0.74 | 14.97 | 0.21 | -36.09 | 1.88 | -0.84 | 8.30 | 0.06 | -34.27 | 1.53 | -0.84 | 2.50 | 0.08 |
| 8 | -14.16 | 1.11 | -0.53 | 4.63 | 0.07 | -21.92 | 3.41 | -0.76 | 21.00 | 0.29 | -37.68 | 2.11 | -0.85 | 5.70 | 0.06 | -33.98 | 1.38 | -0.86 | 3.60 | 0.14 |
| 9 | -14.06 | 1.09 | -0.52 | 7.24 | 0.08 | -22.12 | 3.29 | -0.77 | 21.05 | 0.27 | -37.35 | 2.11 | -0.82 | 7.40 | 0.07 | -33.82 | 1.31 | -0.85 | 4.90 | 0.18 |
| 10 | -16.55 | 1.15 | -0.52 | 5.28 | 0.07 | -24.21 | 3.67 | -0.77 | 3.57 | 0.14 | -45.60 | 2.00 | -0.92 | 8.90 | 0.03 | -31.87 | 1.55 | -0.88 | 1.70 | 0.04 |
| 11 | -15.10 | 0.89 | -0.51 | 4.18 | 0.07 | -25.73 | 2.78 | -0.77 | 11.29 | 0.17 | -37.25 | 0.91 | -0.80 | 6.80 | 0.09 | -35.10 | 1.19 | -0.82 | 6.10 | 0.08 |
| 12 | -16.29 | 1.21 | -0.51 | 4.78 | 0.09 | -26.61 | 3.73 | -0.78 | 4.25 | 0.20 | -44.71 | 2.10 | -0.93 | 8.90 | 0.03 | -34.65 | 1.65 | -0.90 | 1.90 | 0.09 |
| 13 | -11.52 | 0.84 | -0.50 | 2.05 | 0.02 | -14.42 | 2.75 | -0.70 | 4.24 | 0.09 | -32.05 | 0.83 | -0.76 | 3.70 | 0.03 | -27.32 | 1.20 | -0.76 | 2.00 | 0.03 |
| 14 | -16.70 | 1.12 | -0.57 | 8.64 | 0.07 | -23.29 | 3.60 | -0.77 | 5.65 | 0.15 | -45.98 | 1.99 | -0.96 | 12.50 | 0.02 | -32.05 | 1.43 | -0.88 | 3.00 | 0.05 |
| 15 | -15.90 | 1.45 | -0.55 | 5.47 | 0.07 | -21.78 | 4.23 | -0.75 | 13.29 | 0.18 | -40.69 | 2.58 | -0.84 | 10.60 | 0.14 | -28.97 | 1.70 | -0.78 | 4.40 | 0.07 |

ATT – Absolute Effect, ATT% - Relative Effect; S.E. – Standard Error; P. R² - Pseudo R².

**Table S7 –** (continuation)

| **REF** | **Mahalanobis Matching** | | | | | | | | | | | | | | | | | | | |
| --- | --- | --- | --- | --- | --- | --- | --- | --- | --- | --- | --- | --- | --- | --- | --- | --- | --- | --- | --- | --- |
|  | **Between 1996-2002** | | | | | **Between 2002-2008** | | | | | **Indigenous Lands** | | | | | **Quilombola Lands** | | | | |
|  | **ATT** | **S.E.** | **ATT%** | **Bias** | **P. R²** | **ATT** | **S.E.** | **ATT%** | **Bias** | **P. R²** | **ATT** | **S.E.** | **ATT%** | **Bias** | **P. R²** | **ATT** | **S.E.** | **ATT%** | **Bias** | **P. R²** |
| 1 | 1.68 | 0.52 | -0.44 | 1.90 | 0.01 | -5.16 | 1.02 | -0.63 | 1.30 | 0.01 | -12.87 | 0.94 | -0.88 | 2.70 | 0.31 | -0.86 | 0.49 | -0.63 | 1.70 | 0.13 |
| 2 | -0.44 | 0.54 | -0.43 | 1.80 | 0.01 | -6.24 | 1.11 | -0.64 | 2.80 | 0.03 | -12.90 | 1.08 | -0.85 | 0.60 | 0.18 | -4.37 | 0.57 | -0.64 | 7.70 | 0.74 |
| 3 | 0.11 | 0.44 | -0.40 | 2.90 | 0.02 | -6.21 | 1.10 | -0.65 | 1.80 | 0.00 | -14.92 | 0.98 | -0.89 | 5.10 | 0.41 | -4.72 | 0.52 | -0.59 | 5.10 | 0.77 |
| 4 | 1.68 | 0.58 | -0.40 | 0.80 | 0.00 | -5.65 | 1.18 | -0.65 | 1.00 | 0.00 | -13.14 | 1.06 | -0.89 | 3.90 | 0.42 | -0.67 | 0.64 | -0.56 | 0.20 | 0.02 |
| 5 | 1.66 | 0.50 | -0.42 | 1.70 | 0.00 | -5.54 | 1.08 | -0.63 | 1.10 | 0.00 | -11.63 | 0.88 | -0.87 | 1.50 | 0.32 | -0.86 | 0.49 | -0.63 | 2.40 | 0.07 |
| 6 | 0.15 | 0.41 | -0.45 | 4.90 | 0.02 | -5.48 | 1.07 | -0.63 | 5.30 | 0.01 | -14.52 | 0.93 | -0.92 | 6.50 | 0.44 | -4.87 | 0.47 | -0.74 | 8.00 | 0.77 |
| 7 | 2.52 | 0.55 | -0.42 | 4.80 | 0.02 | -4.63 | 1.08 | -0.63 | 1.70 | 0.01 | -13.55 | 0.98 | -0.90 | 3.10 | 0.29 | -0.48 | 0.52 | -0.63 | 2.60 | 0.05 |
| 8 | 1.44 | 0.43 | -0.44 | 4.60 | 0.04 | -5.59 | 1.12 | -0.63 | 2.60 | 0.01 | -12.58 | 1.01 | -0.89 | 4.00 | 0.31 | -4.66 | 0.57 | -0.71 | 29.20 | 0.71 |
| 9 | 1.33 | 0.42 | -0.43 | 9.90 | 0.04 | -5.05 | 1.10 | -0.62 | 4.60 | 0.02 | -12.84 | 0.96 | -0.90 | 5.70 | 0.34 | -5.22 | 0.54 | -0.71 | 29.50 | 0.71 |
| 10 | -0.79 | 0.44 | -0.43 | 5.00 | 0.03 | -5.10 | 1.11 | -0.63 | 3.50 | 0.02 | -21.20 | 1.30 | -0.88 | 8.60 | 0.59 | -5.57 | 0.58 | -0.49 | 34.40 | 0.74 |
| 11 | -0.77 | 0.58 | -0.42 | 3.20 | 0.01 | -5.63 | 1.10 | -0.64 | 1.40 | 0.01 | -15.91 | 1.22 | -0.88 | 5.70 | 0.59 | -1.34 | 0.51 | -0.49 | 4.40 | 0.06 |
| 12 | 0.09 | 0.47 | -0.41 | 3.20 | 0.03 | -5.79 | 1.15 | -0.63 | 2.70 | 0.02 | -16.61 | 1.20 | -0.91 | 7.10 | 0.54 | -6.14 | 0.45 | -0.58 | 38.70 | 0.75 |
| 13 | 1.25 | 0.51 | -0.42 | 1.40 | 0.00 | -3.99 | 1.10 | -0.61 | 1.50 | 0.00 | -13.61 | 0.87 | -0.89 | 4.80 | 0.38 | -1.10 | 0.48 | -0.67 | 2.60 | 0.01 |
| 14 | -0.98 | 0.42 | -0.47 | 9.30 | 0.03 | -4.62 | 1.10 | -0.63 | 5.00 | 0.03 | -21.58 | 1.18 | -0.92 | 12.70 | 0.58 | -6.09 | 0.52 | -0.67 | 35.10 | 0.73 |
| 15 | -2.35 | 0.75 | -0.46 | 4.50 | 0.01 | -5.88 | 1.20 | -0.64 | 2.50 | 0.01 | -22.17 | 2.17 | -0.91 | 4.60 | 0.03 | -7.41 | 0.90 | -0.82 | 33.50 | 0.22 |

ATT – Absolute Effect, ATT% - Relative Effect; S.E. – Standard Error; P. R² - Pseudo R².
