# Supplementary material for: The Role of Protected Areas in the Avoidance of Anthropogenic Conversion in a High Pressure Region: A Matching Method Analysis in the Core Region of the Brazilian Cerrado
Source: PLoS One. 2015 Jul 29;10(7):e0132582. doi: 10.1371/journal.pone.0132582 (PMC4519267; doi:10.1371/journal.pone.0132582)
Supplement: S7 Table — (DOCX) [file pone.0132582.s009.docx]

**Table S7 –** Values of Pmax for levels of Г for the best data group.

| **Г** | Strictly Protected | | | | Sustainable Use | | | | Federal Sphere | | | State Sphere | | | | Larger Size | | | | Smaller Size | | | |
| --- | --- | --- | --- | --- | --- | --- | --- | --- | --- | --- | --- | --- | --- | --- | --- | --- | --- | --- | --- | --- | --- | --- | --- |
|  | 1986 | 1996 | 2002 | 2008 | 1986 | 1996 | 2002 | 2008 | 1986 | 2002 | 2008 | 1986 | 1996 | 2002 | 2008 | 1986 | 1996 | 2002 | 2008 | 1986 | 1996 | 2002 | 2008 |
| 1.0 | NS | NS | NS | NS | NS | NS | NS | NS | NS | NS | NS | NS | NS | NS | NS | NS | NS | NS | NS | NS | NS | NS | NS |
| 1.5 | NS | NS | NS | NS | NS | NS | NS | NS | NS | NS | NS | NS | NS | NS | NS | NS | NS | NS | NS | NS | NS | NS | NS |
| 1.8 | NS | NS | NS | NS | NS | NS | <0.05 | NS | NS | NS | NS | NS | NS | <0.05 | <0.05 | NS | NS | NS | <0.05 | NS | <0.05 | NS | NS |
| 2.0 | NS | NS | NS | NS | NS | NS |  | NS | NS | <0.05 | NS | NS | NS |  |  | NS | NS | <0.05 |  | NS |  | NS | NS |
| 2.5 | NS | NS | NS | NS | NS | NS |  | NS | NS |  | NS | NS | NS |  |  | NS | NS |  |  | NS |  | <0.05 | NS |
| 2.8 | NS | NS | NS | <0.05 | NS | <0.05 |  | NS | NS |  | NS | NS | NS |  |  | NS | NS |  |  | NS |  |  | NS |
| 3.0 | NS | NS | NS |  | NS |  |  | NS | NS |  | NS | NS | NS |  |  | NS | NS |  |  | NS |  |  | NS |
| 3.3 | NS | NS | <0.05 |  | NS |  |  | NS | NS |  | NS | NS | NS |  |  | NS | NS |  |  | NS |  |  | <0.05 |
| 3.5 | NS | NS |  |  | <0.05 |  |  | NS | NS |  | NS | NS | NS |  |  | NS | NS |  |  | NS |  |  |  |
| 4.0 | NS | NS |  |  |  |  |  | NS | NS |  | NS | NS | NS |  |  | NS | NS |  |  | NS |  |  |  |
| 4.5 | NS | NS |  |  |  |  |  | NS | NS |  | NS | NS | NS |  |  | NS | NS |  |  | NS |  |  |  |
| 5.0 | NS | NS |  |  |  |  |  | NS | NS |  | NS | NS | NS |  |  | NS | NS |  |  | NS |  |  |  |

**Table S7 –** (continuation)

| **Г** | Before  1986 | Between  1986-1996 | Between  1996-2002 | Between  2002-2008 | Indigenous Lands | Quilombola Lands |
| --- | --- | --- | --- | --- | --- | --- |
| 1.0 | NS | NS | NS | NS | NS | NS |
| 1.5 | NS | NS | NS | NS | NS | NS |
| 1.8 | NS | NS | <0.05 | NS | NS | NS |
| 2.0 | NS | NS |  | NS | NS | NS |
| 2.5 | NS | NS |  | <0.05 | NS | NS |
| 2.8 | NS | NS |  |  | NS | NS |
| 3.0 | NS | NS |  |  | NS | NS |
| 3.3 | NS | NS |  |  | NS | NS |
| 3.5 | NS | NS |  |  | NS | NS |
| 4.0 | NS | NS |  |  | NS | NS |
| 4.5 | NS | NS |  |  | NS | <0.05 |
| 5.0 | NS | NS |  |  | NS |  |
