# Supplementary material for: The Role of Protected Areas in the Avoidance of Anthropogenic Conversion in a High Pressure Region: A Matching Method Analysis in the Core Region of the Brazilian Cerrado
Source: PLoS One. 2015 Jul 29;10(7):e0132582. doi: 10.1371/journal.pone.0132582 (PMC4519267; doi:10.1371/journal.pone.0132582)
Supplement: S8 Table — (DOCX) [file pone.0132582.s010.docx]

**Table S8 –** Results for the non-buffer and buffer groups.

| **Group/Subgroup** | **Buffer Group** | | | | | | | | | | **Non-Buffer Group** | | | | | | | | | |
| --- | --- | --- | --- | --- | --- | --- | --- | --- | --- | --- | --- | --- | --- | --- | --- | --- | --- | --- | --- | --- |
|  | **ATT** | | **ATT%** | | **S.E.** | | **Bias** | | **P.R²** | | **ATT** | | **ATT%** | | **S.E.** | | **Bias** | | **P.R²** | |
|  | **Mean** | **Std. Dev.** | **Mean** | **Std. Dev.** | **Mean** | **Std. Dev.** | **Mean** | **Std. Dev.** | **Mean** | **Std. Dev.** | **Mean** | **Std. Dev.** | **Mean** | **Std. Dev.** | **Mean** | **Std. Dev.** | **Mean** | **Std. Dev.** | **Mean** | **Std. Dev.** |
| **Protected Areas** |  |  |  |  |  |  |  |  |  |  |  |  |  |  |  |  |  |  |  |  |
| All Protected Areas | -15.04 | 1.63 | -0.53 | 0.02 | 0.98 | 0.17 | 4.16 | 2.09 | 0.03 | 0.02 | -13.79 | 1.21 | -0.49 | 0.02 | 1.05 | 0.21 | 4.53 | 2.22 | 0.02 | 0.01 |
| **Restriction Group** |  |  |  |  |  |  |  |  |  |  |  |  |  |  |  |  |  |  |  |  |
| Strictly Protected | -25.87 | 0.92 | -0.90 | 0.01 | 1.91 | 0.28 | 4.29 | 1.74 | 0.05 | 0.03 | -25.90 | 1.00 | -0.90 | 0.01 | 1.88 | 0.25 | 3.66 | 1.24 | 0.03 | 0.02 |
| Sustainable Use | -3.39 | 2.47 | -0.43 | 0.03 | 2.59 | 1.84 | 6.49 | 4.18 | 0.22 | 0.05 | -3.19 | 2.04 | -0.38 | 0.02 | 1.69 | 0.37 | 6.77 | 3.86 | 0.10 | 0.04 |
| **Sphere Group** |  |  |  |  |  |  |  |  |  |  |  |  |  |  |  |  |  |  |  |  |
| Federal Sphere | -19.35 | 2.83 | -0.68 | 0.05 | 1.13 | 0.26 | 4.13 | 2.03 | 0.05 | 0.03 | -18.32 | 2.07 | -0.63 | 0.03 | 1.21 | 0.28 | 4.17 | 2.15 | 0.03 | 0.02 |
| State Sphere | -13.28 | 1.23 | -0.44 | 0.03 | 1.33 | 0.18 | 5.19 | 2.45 | 0.09 | 0.03 | -12.63 | 1.05 | -0.40 | 0.02 | 1.41 | 0.22 | 5.93 | 2.91 | 0.05 | 0.02 |
| **Size Group** |  |  |  |  |  |  |  |  |  |  |  |  |  |  |  |  |  |  |  |  |
| Larger Size | -14.31 | 1.62 | -0.52 | 0.02 | 1.03 | 0.16 | 4.14 | 2.11 | 0.06 | 0.02 | -12.96 | 1.25 | -0.48 | 0.02 | 1.07 | 0.20 | 4.54 | 2.28 | 0.03 | 0.01 |
| Smaller Size | -21.96 | 3.28 | -0.75 | 0.02 | 3.29 | 0.42 | 8.40 | 6.25 | 0.16 | 0.07 | -22.38 | 2.40 | -0.73 | 0.02 | 3.47 | 0.41 | 10.11 | 5.84 | 0.09 | 0.04 |
| **Age Group** |  |  |  |  |  |  |  |  |  |  |  |  |  |  |  |  |  |  |  |  |
| Before 1986 | -37.83 | 4.88 | -0.84 | 0.07 | 1.57 | 0.55 | 6.22 | 3.31 | 0.05 | 0.03 | -36.71 | 3.73 | -0.80 | 0.02 | 1.85 | 0.57 | 5.57 | 2.45 | 0.03 | 0.01 |
| Between 1986-1996 | -32.26 | 2.35 | -0.82 | 0.05 | 1.35 | 0.22 | 3.06 | 1.67 | 0.06 | 0.04 | -29.98 | 1.76 | -0.80 | 0.02 | 1.43 | 0.25 | 5.89 | 3.28 | 0.03 | 0.02 |
| Between 1996-2002 | 0.44 | 1.30 | -0.43 | 0.02 | 0.50 | 0.09 | 3.99 | 2.58 | 0.02 | 0.01 | 1.68 | 0.79 | -0.38 | 0.03 | 0.48 | 0.12 | 4.07 | 2.29 | 0.01 | 0.01 |
| Between 2002-2008 | -5.37 | 0.60 | -0.63 | 0.01 | 1.11 | 0.04 | 2.59 | 1.38 | 0.01 | 0.01 | -4.74 | 0.67 | -0.62 | 0.02 | 1.09 | 0.04 | 2.50 | 1.39 | 0.01 | 0.01 |
| **Other Areas** |  |  |  |  |  |  |  |  |  |  |  |  |  |  |  |  |  |  |  |  |
| Indigenous Lands | -15.33 | 3.40 | -0.89 | 0.02 | 1.12 | 0.31 | 5.11 | 2.86 | 0.38 | 0.15 | -11.94 | 2.47 | -0.88 | 0.03 | 1.09 | 0.32 | 4.09 | 2.73 | 0.28 | 0.12 |
| Quilombola Lands | -3.62 | 2.35 | -0.64 | 0.09 | 0.55 | 0.10 | 15.67 | 14.75 | 0.43 | 0.33 | -2.10 | 1.51 | -0.60 | 0.09 | 0.50 | 0.18 | 9.68 | 9.19 | 0.32 | 0.27 |

ATT – Absolute Effect, ATT% - Relative Effect; S.E. – Standard Error; P. R² - Pseudo R².

**Table S8** – (continuation)

| **Group/Subgroup** | **Wilcoxon Paired Test - Buffer x Non-buffer** | | | | | | | | | |
| --- | --- | --- | --- | --- | --- | --- | --- | --- | --- | --- |
|  | **ATT** | | **ATT%** | | **S.E.** | | **Bias** | | **P.R²** | |
|  | **Z** | ***p*** | **Z** | ***p*** | **Z** | ***p*** | **Z** | ***p*** | **Z** | ***p*** |
| **Protected Areas** |  |  |  |  |  |  |  |  |  |  |
| All Protected Areas | -2.302 | 0.021 | -3.694 | <0.001 | -0.913 | 0.361 | -0.394 | 0.694 | 1.953 | 0.051 |
| **Restriction Group** |  |  |  |  |  |  |  |  |  |  |
| Strictly Protected | 0.104 | 0.917 | 0.173 | 0.863 | 0.187 | 0.852 | 0.892 | 0.373 | 1.468 | 0.142 |
| Sustainable Use | -0.187 | 0.852 | -3.727 | <0.001 | 0.477 | 0.633 | -0.311 | 0.756 | 4.158 | <0.001 |
| **Government Sphere Group** |  |  |  |  |  |  |  |  |  |  |
| Federal Sphere | -0.850 | 0.395 | -2.520 | 0.012 | -0.540 | 0.589 | -0.104 | 0.917 | 2.032 | 0.042 |
| State Sphere | -1.659 | 0.097 | -3.357 | <0.001 | -0.954 | 0.340 | -0.767 | 0.443 | 3.073 | 0.002 |
| **Size Group** |  |  |  |  |  |  |  |  |  |  |
| Larger Size | -2.509 | 0.012 | -3.631 | <0.001 | -0.353 | 0.724 | -0.436 | 0.663 | 2.587 | 0.010 |
| Smaller Size | 0.187 | 0.852 | -2.095 | 0.036 | -1.369 | 0.171 | -0.850 | 0.395 | 3.160 | 0.002 |
| **Age Group** |  |  |  |  |  |  |  |  |  |  |
| Before 1986 | -0.767 | 0.443 | -1.586 | 0.113 | -1.390 | 0.165 | 0.602 | 0.547 | 1.347 | 0.178 |
| Between 1986-1996 | -2.510 | 0.012 | -0.944 | 0.345 | -0.560 | 0.575 | -2.262 | 0.024 | 2.400 | 0.016 |
| Between 1996-2002 | -2.427 | 0.015 | -3.877 | <0.001 | 1.394 | 0.163 | -0.125 | 0.901 | 1.199 | 0.231 |
| Between 2002-2008 | -2.386 | 0.017 | -1.660 | 0.097 | 1.025 | 0.305 | 0.353 | 0.724 | 1.479 | 0.139 |
| **Other Areas** |  |  |  |  |  |  |  |  |  |  |
| Indigenous Lands | -3.132 | 0.002 | -1.173 | 0.241 | 0.685 | 0.494 | 1.245 | 0.213 | 1.952 | 0.051 |
| Quilombola Lands | -2.095 | 0.036 | -0.977 | 0.329 | 1.391 | 0.164 | 0.788 | 0.430 | 1.682 | 0.093 |

ATT – Absolute Effect, ATT% - Relative Effect; S.E. – Standard Error; P. R² - Pseudo R².
