# Supplementary material for: The Role of Protected Areas in the Avoidance of Anthropogenic Conversion in a High Pressure Region: A Matching Method Analysis in the Core Region of the Brazilian Cerrado
Source: PLoS One. 2015 Jul 29;10(7):e0132582. doi: 10.1371/journal.pone.0132582 (PMC4519267; doi:10.1371/journal.pone.0132582)
Supplement: S9 Table — (DOCX) [file pone.0132582.s011.docx]

**Table S9 –** Statistics for the sample units in the control group effectively used to obtain the effectiveness of protected areas.

| **Groups/subgroups** | **S.U.** | **Mean** | **Median** | **Std. Dev.** | **S.E.** | **Kurtosis** | **Skewness** |
| --- | --- | --- | --- | --- | --- | --- | --- |
| **Type Group** |  |  |  |  |  |  |  |
| Protected Areas | 15122 | 16.97 | 9.40 | 19.75 | 0.18 | 5.03 | 1.61 |
| Indigenous Lands | 1213 | 18.09 | 14.22 | 15.30 | 0.86 | 6.13 | 1.52 |
| Quilombola Lands | 2082 | 8.03 | 4.41 | 9.26 | 0.20 | 5.97 | 1.61 |
| **Restriction Group** |  |  |  |  |  |  |  |
| Strictly Protected | 3656 | 40.66 | 40.40 | 24.43 | 0.49 | 2.03 | 0.10 |
| Sustainable Use | 11285 | 10.87 | 6.59 | 12.44 | 0.13 | 8.50 | 2.04 |
| **Sphere Group** |  |  |  |  |  |  |  |
| Federal Sphere | 6004 | 29.08 | 21.55 | 22.70 | 0.35 | 2.79 | 0.87 |
| State Sphere | 9317 | 10.72 | 5.25 | 14.30 | 0.16 | 8.93 | 2.29 |
| **Size Group** |  |  |  |  |  |  |  |
| Larger Size | 13334 | 16.73 | 9.04 | 19.75 | 0.18 | 5.10 | 1.64 |
| Smaller Size | 1305 | 25.10 | 21.71 | 17.95 | 0.95 | 4.55 | 1.12 |
| **Age Group** |  |  |  |  |  |  |  |
| Before 1986 | 1856 | 45.05 | 46.46 | 24.67 | 0.59 | 2.13 | -0.21 |
| Between 1986-1996 | 1595 | 39.25 | 38.69 | 21.59 | 0.82 | 2.34 | 0.22 |
| Between 1996-2002 | 9723 | 10.05 | 6.27 | 11.00 | 0.11 | 6.96 | 1.75 |
| Between 2002-2008 | 1320 | 14.20 | 12.79 | 8.12 | 0.39 | 3.33 | 0.78 |

S.U. - sampling units (control group), Std. Dev. - Standard Deviation, S.E - Standard Error.

*Mean, median, Standard Deviation and Standard Error - ha/km².
