# Supplementary material for: The Role of Protected Areas in the Avoidance of Anthropogenic Conversion in a High Pressure Region: A Matching Method Analysis in the Core Region of the Brazilian Cerrado
Source: PLoS One. 2015 Jul 29;10(7):e0132582. doi: 10.1371/journal.pone.0132582 (PMC4519267; doi:10.1371/journal.pone.0132582)
Supplement: S10 Table — (DOCX) [file pone.0132582.s012.docx]

**Table S10 –** Results for the restriction groups with respect to government sphere, age, and size subgroups.

| **Groups/subgroups** | **PA Units** | **S.U.** | | **ATT** | | **S.E.** | | **ATT%** | | **Bias** | | **P. R²** | |
| --- | --- | --- | --- | --- | --- | --- | --- | --- | --- | --- | --- | --- | --- |
|  |  |  |  |  |  |  |  |  |  |  |  |  |  |
|  |  | **On** | **Total** | **Mean** | **Std. Dev.** | **Mean** | **Std. Dev.** | **Mean** | **Std. Dev.** | **Mean** | **Std. Dev.** | **Mean** | **Std. Dev.** |
| **All PAs** |  |  |  |  |  |  |  |  |  |  |  |  |  |
| Strictly Protected | 15 | 2765 | 2917 | -25.87 | 0.92 | 1.91 | 0.28 | -0.90 | 0.01 | 4.29 | 1.74 | 0.05 | 0.03 |
| Sustainable Use | 24 | 10280 | 10735 | -3.39 | 2.47 | 2.59 | 1.84 | -0.43 | 0.03 | 6.49 | 4.18 | 0.22 | 0.05 |
| **Government Sphere Group** |  |  |  |  |  |  |  |  |  |  |  |  |  |
| Federal Sphere |  |  |  |  |  |  |  |  |  |  |  |  |  |
| Strictly Protected | 4 | 1904 | 2032 | -32.63 | 4.03 | 1.82 | 0.56 | -0.95 | 0.02 | 3.26 | 2.09 | 0.03 | 0.03 |
| Sustainable Use | 11 | 3072 | 3429 | 0.10 | 3.66 | 3.77 | 3.17 | -0.51 | 0.08 | 7.69 | 5.73 | 0.28 | 0.07 |
| State Sphere |  |  |  |  |  |  |  |  |  |  |  |  |  |
| Strictly Protected | 11 | 860 | 885 | -22.42 | 1.80 | 2.31 | 0.25 | -0.80 | 0.01 | 4.50 | 2.54 | 0.11 | 0.05 |
| Sustainable Use | 13 | 7195 | 7306 | -8.83 | 2.54 | 1.65 | 0.54 | -0.40 | 0.03 | 4.05 | 1.96 | 0.14 | 0.07 |
| **Size Group** |  |  |  |  |  |  |  |  |  |  |  |  |  |
| Larger Size |  |  |  |  |  |  |  |  |  |  |  |  |  |
| Striclyt Protected | 5 | 2550 | 2676 | -29.83 | 0.74 | 1.66 | 0.32 | -0.91 | 0.01 | 3.96 | 2.16 | 0.08 | 0.04 |
| Sustainable Use | 14 | 10028 | 10460 | -1.21 | 3.20 | 1.61 | 0.41 | -0.42 | 0.03 | 6.06 | 3.75 | 0.22 | 0.05 |
| Smaller Size |  |  |  |  |  |  |  |  |  |  |  |  |  |
| Strictly Protected | 10 | 214 | 241 | -17.39 | 3.39 | 6.37 | 0.94 | -0.85 | 0.02 | 7.09 | 4.09 | 0.13 | 0.06 |
| Sustainable Use | 10 | 252 | 275 | -11.00 | 8.69 | 3.82 | 1.66 | -0.67 | 0.06 | 8.04 | 7.24 | 0.25 | 0.12 |
| **Age Group** |  |  |  |  |  |  |  |  |  |  |  |  |  |
| Before 1986 |  |  |  |  |  |  |  |  |  |  |  |  |  |
| Strictly Protected | 5 | 2032 | 2172 | -43.16 | 2.48 | 1.40 | 0.52 | -0.95 | 0.02 | 5.21 | 3.21 | 0.03 | 0.02 |
| Sustainable Use | 5 | 246 | 365 | 11.78 | 9.63 | 10.42 | 10.3 | 0.21 | 0.15 | 19.03 | 17.82 | 0.85 | 0.24 |
| Between 1986-1996 |  |  |  |  |  |  |  |  |  |  |  |  |  |
| Striclyt Protected | 4 | 504 | 511 | -36.75 | 2.84 | 1.45 | 0.34 | -0.95 | 0.02 | 1.68 | 1.15 | 0.03 | 0.05 |
| Sustainable Use | 4 | 301 | 368 | -24.98 | 5.52 | 2.18 | 0.27 | -0.62 | 0.07 | 5.00 | 3.11 | 0.34 | 0.06 |
| Between 1996-2002 |  |  |  |  |  |  |  |  |  |  |  |  |  |
| Strictly Protected | 4 | 39 | 42 | -11.58 | 2.69 | 2.87 | 0.20 | -0.59 | 0.07 | 5.61 | 3.64 | 0.05 | 0.06 |
| Sustainable Use | 11 | 9491 | 9760 | 0.50 | 1.31 | 0.50 | 0.09 | -0.43 | 0.02 | 3.99 | 2.60 | 0.02 | 0.01 |
| Between 2002-2008 |  |  |  |  |  |  |  |  |  |  |  |  |  |
| Strictly Protected | 2 | 190 | 192 | 0.25 | 1.16 | 2.21 | 0.10 | -0.31 | 0.02 | 4.55 | 2.40 | 0.10 | 0.07 |
| Sustainable Use | 4 | 242 | 242 | -9.84 | 0.72 | 0.68 | 0.06 | -0.88 | 0.01 | 1.54 | 1.36 | 0.03 | 0.04 |

S.U. - sampling units (On – average number of S. U. on support); ATT – Absolute Effect, ATT% - Relative Effect; S.E - Standard Error; Mean - Mean for the 15 Best Models; Std. Dev. - Standard Deviation for the 15 Best Models, P. R^2^ – Pseudo R^2^. * No data.

**Table S10** – (continuation)

| **Groups/subgroups** |  | **Wilcoxon Paired Test** | | |
| --- | --- | --- | --- | --- |
|  | **ATT** | | **ATT%** | |
|  | **Z** | ***p*** | **Z** | ***p*** |
| **All PAs** |  |  |  |  |
| Strictly Protected | - | - | - | - |
| Sustainable Use | -4.66 | <0.001 | -4.67 | <0.001 |
| **Government Sphere Group** |  |  |  |  |
| Federal Sphere |  |  |  |  |
| Strictly Protected | - | - | - | - |
| Sustainable Use | -4.66 | <0.001 | -4.67 | <0.001 |
| State Sphere |  |  |  |  |
| Strictly Protected | - | - | - | - |
| Sustainable Use | -4.66 | <0.001 | -4.67 | <0.001 |
| **Size Group** |  |  |  |  |
| Larger Size |  |  |  |  |
| Strictly Protected | - | - | - | - |
| Sustainable Use | -4.66 | <0.001 | -4.67 | <0.001 |
| Smaller Size |  |  |  |  |
| Strictly Protected | - | - | - | - |
| Sustainable Use | -2.26 | 0.024 | -4.67 | <0.001 |
| **Age Group** |  |  |  |  |
| Before 1986 |  |  |  |  |
| Strictly Protected | - | - | - | - |
| Sustainable Use | -4.66 | <0.001 | -4.67 | <0.001 |
| Between 1986-1996 |  |  |  |  |
| Strictly Protected | - | - | - | - |
| Sustainable Use | -4.42 | <0.001 | -4.67 | <0.001 |
| Between 1996-2002 |  |  |  |  |
| Strictly Protected | - | - | - | - |
| Sustainable Use | -4.66 | <0.001 | -4.54 | <0.001 |
| Between 2002-2008 |  |  |  |  |
| Strictly Protected | - | - | - | - |
| Sustainable Use | 4.66 | <0.001 | 4.66 | <0.001 |
