# Supplementary material for: The Role of Protected Areas in the Avoidance of Anthropogenic Conversion in a High Pressure Region: A Matching Method Analysis in the Core Region of the Brazilian Cerrado
Source: PLoS One. 2015 Jul 29;10(7):e0132582. doi: 10.1371/journal.pone.0132582 (PMC4519267; doi:10.1371/journal.pone.0132582)
Supplement: S11 Table — (DOCX) [file pone.0132582.s013.docx]

**Table S11 –** Results for the government sphere groups with respect to restriction, age, and size subgroups.

| **Groups/subgroups** | **PA Units** | **S.U.** | | **ATT** | | **S.E.** | | **ATT%** | | **Bias** | | **P. R²** | |
| --- | --- | --- | --- | --- | --- | --- | --- | --- | --- | --- | --- | --- | --- |
|  |  |  |  |  |  |  |  |  |  |  |  |  |  |
|  |  | **On** | **Total** | **Mean** | **Std. Dev.** | **Mean** | **Std. Dev.** | **Mean** | **Std. Dev.** | **Mean** | **Std. Dev.** | **Mean** | **Std. Dev.** |
| **All PAs** |  |  |  |  |  |  |  |  |  |  |  |  |  |
| Federal | 15 | 4977 | 5461 | -19.35 | 2.83 | 1.13 | 0.26 | -0.68 | 0.05 | 4.13 | 2.03 | 0.05 | 0.03 |
| Stadual | 24 | 8056 | 8191 | -13.28 | 1.23 | 1.33 | 0.18 | -0.44 | 0.03 | 5.19 | 2.45 | 0.09 | 0.03 |
| **Restriction Group** |  |  |  |  |  |  |  |  |  |  |  |  |  |
| Strictly Protected |  |  |  |  |  |  |  |  |  |  |  |  |  |
| Federal | 4 | 1904 | 2032 | -32.63 | 4.03 | 1.82 | 0.56 | -0.95 | 0.02 | 3.26 | 2.09 | 0.03 | 0.03 |
| State | 11 | 860 | 885 | -22.42 | 1.80 | 2.31 | 0.25 | -0.80 | 0.01 | 4.50 | 2.54 | 0.11 | 0.05 |
| Sustainable Use |  |  |  |  |  |  |  |  |  |  |  |  |  |
| Federal | 11 | 3072 | 3429 | 0.10 | 3.66 | 3.77 | 3.17 | -0.51 | 0.08 | 7.69 | 5.73 | 0.28 | 0.07 |
| Stadual | 13 | 7195 | 7306 | -8.83 | 2.54 | 1.65 | 0.54 | -0.40 | 0.03 | 4.05 | 1.96 | 0.14 | 0.07 |
| **Size Group** |  |  |  |  |  |  |  |  |  |  |  |  |  |
| Larger Size |  |  |  |  |  |  |  |  |  |  |  |  |  |
| Federal | 9 | 4871 | 5346 | -19.63 | 3.04 | 1.21 | 0.26 | -0.67 | 0.05 | 4.19 | 2.04 | 0.07 | 0.03 |
| State | 10 | 7694 | 7790 | -7.15 | 0.73 | 1.05 | 0.11 | -0.43 | 0.03 | 4.24 | 2.09 | 0.08 | 0.04 |
| Smaller Size |  |  |  |  |  |  |  |  |  |  |  |  |  |
| Federal | 6 | 106 | 115 | -24.90 | 3.77 | 3.07 | 1.69 | -0.85 | 0.03 | 5.95 | 5.83 | 0.23 | 0.16 |
| State | 14 | 360 | 401 | -21.73 | 3.58 | 3.78 | 0.42 | -0.72 | 0.03 | 8.03 | 5.39 | 0.19 | 0.08 |
| **Age Group** |  |  |  |  |  |  |  |  |  |  |  |  |  |
| Before 1986 |  |  |  |  |  |  |  |  |  |  |  |  |  |
| Federal | 7 | 2121 | 2352 | -37.77 | 5.22 | 1.61 | 0.55 | -0.84 | 0.07 | 6.12 | 3.38 | 0.08 | 0.05 |
| State | 3 | 158 | 185 | -37.60 | 4.71 | 3.08 | 0.72 | -0.92 | 0.04 | 10.91 | 9.06 | 0.35 | 0.14 |
| Between 1986-1996 |  |  |  |  |  |  |  |  |  |  |  |  |  |
| Federal* |  |  |  |  |  |  |  |  |  |  |  |  |  |
| Stadual | 8 | 804 | 879 | -32.26 | 2.35 | 1.35 | 0.22 | -0.82 | 0.05 | 3.06 | 1.67 | 0.06 | 0.04 |
| Between 1996-2002 |  |  |  |  |  |  |  |  |  |  |  |  |  |
| Federal | 5 | 2673 | 2925 | -4.81 | 2.68 | 0.79 | 0.12 | -0.54 | 0.06 | 3.77 | 2.04 | 0.04 | 0.03 |
| State | 10 | 6845 | 6877 | 2.40 | 0.84 | 0.56 | 0.10 | -0.39 | 0.03 | 4.90 | 2.95 | 0.03 | 0.02 |
| Between 2002-2008 |  |  |  |  |  |  |  |  |  |  |  |  |  |
| Federal | 3 | 184 | 184 | -10.46 | 1.24 | 0.85 | 0.07 | -0.86 | 0.02 | 1.22 | 1.10 | 0.03 | 0.04 |
| Stadual | 3 | 248 | 250 | -1.68 | 0.95 | 1.75 | 0.07 | -0.46 | 0.02 | 3.86 | 1.85 | 0.02 | 0.02 |

S.U. - sampling units (On – average number of S. U. on support); ATT – Absolute Effect, ATT% - Relative Effect; S.E - Standard Error; Mean - Mean for the 15 Best Models; Std. Dev. - Standard Deviation for the 15 Best Models, P. R^2^ – Pseudo R^2^. * No data.

**Table S11** – (continuation)

| **Groups/subgroups** |  | **Wilcoxon Paired Test** | | |
| --- | --- | --- | --- | --- |
|  | **ATT** | | **ATT%** | |
|  | **Z** | ***p*** | **Z** | ***p*** |
| **All PAs** |  |  |  |  |
| Federal | - | - | - | - |
| Stadual | -4.583 | <0.001 | -4.67 | <0.001 |
| **Restriction Group** |  |  |  |  |
| Strictly Protected |  |  |  |  |
| Federal | - | - | - | - |
| State | -4.625 | <0.001 | -4.67 | <0.001 |
| Sustainable Use |  |  |  |  |
| Federal | - | - | - | - |
| Stadual | 4.583 | <0.001 | -3.63 | <0.001 |
| **Size Group** |  |  |  |  |
| Larger Size |  |  |  |  |
| Federal | - | - | - | - |
| State | -4.666 | <0.001 | -4.67 | <0.001 |
| Smaller Size |  |  |  |  |
| Federal | - | - | - | - |
| State | -2.136 | 0.033 | -4.67 | <0.001 |
| **Age Group** |  |  |  |  |
| Before 1986 |  |  |  |  |
| Federal | - | - | - | - |
| State | -0.353 | 0.724 | 2.924 | 0.004 |
| Between 1986-1996 |  |  |  |  |
| Federal* | - | - | - | - |
| Stadual | - | - | - | - |
| Between 1996-2002 |  |  |  |  |
| Federal | - | - | - | - |
| State | -4.666 | <0.001 | -4.67 | <0.001 |
| Between 2002-2008 |  |  |  |  |
| Federal | - | - | - | - |
| Stadual | -4.666 | <0.001 | -4.67 | <0.001 |
