# Supplementary material for: The Role of Protected Areas in the Avoidance of Anthropogenic Conversion in a High Pressure Region: A Matching Method Analysis in the Core Region of the Brazilian Cerrado
Source: PLoS One. 2015 Jul 29;10(7):e0132582. doi: 10.1371/journal.pone.0132582 (PMC4519267; doi:10.1371/journal.pone.0132582)
Supplement: S12 Table — (DOCX) [file pone.0132582.s014.docx]

**Table S12 –** Results for the size groups with respect to restriction, government sphere, and age.

| **Groups/subgroups** | **PA Units** | **S.U.** | | **ATT** | | **S.E.** | | **ATT%** | | **Bias** | | **P. R²** | |
| --- | --- | --- | --- | --- | --- | --- | --- | --- | --- | --- | --- | --- | --- |
|  |  |  |  |  |  |  |  |  |  |  |  |  |  |
|  |  | **On** | **Total** | **Mean** | **Std. Dev.** | **Mean** | **Std. Dev.** | **Mean** | **Std. Dev.** | **Mean** | **Std. Dev.** | **Mean** | **Std. Dev.** |
| **All PAs** |  |  |  |  |  |  |  |  |  |  |  |  |  |
| Larger Size | 19 | 12579 | 13136 | -14.31 | 1.62 | 1.03 | 0.16 | -0.52 | 0.02 | 4.14 | 2.11 | 0.06 | 0.02 |
| Smaller Size | 20 | 466 | 516 | -21.96 | 3.28 | 3.29 | 0.42 | -0.75 | 0.02 | 8.40 | 6.25 | 0.16 | 0.07 |
| **Restriction Group** |  |  |  |  |  |  |  |  |  |  |  |  |  |
| Strictly Protected |  |  |  |  |  |  |  |  |  |  |  |  |  |
| Larger Size | 5 | 2550 | 2676 | -29.83 | 0.74 | 1.66 | 0.32 | -0.91 | 0.01 | 3.96 | 2.16 | 0.08 | 0.04 |
| Smaller Size | 10 | 214 | 241 | -17.39 | 3.39 | 6.37 | 0.94 | -0.85 | 0.02 | 7.09 | 4.09 | 0.13 | 0.06 |
| Sustainable Use |  |  |  |  |  |  |  |  |  |  |  |  |  |
| Larger Size | 14 | 10028 | 10460 | -1.21 | 3.20 | 1.61 | 0.41 | -0.42 | 0.03 | 6.06 | 3.75 | 0.22 | 0.05 |
| Smaller Size | 10 | 252 | 275 | -11.00 | 8.69 | 3.82 | 1.66 | -0.67 | 0.06 | 8.04 | 7.24 | 0.25 | 0.12 |
| **Government Sphere Group** |  |  |  |  |  |  |  |  |  |  |  |  |  |
| Federal Sphere |  |  |  |  |  |  |  |  |  |  |  |  |  |
| Larger Size | 9 | 4871 | 5346 | -19.63 | 3.04 | 1.21 | 0.26 | -0.67 | 0.05 | 4.19 | 2.04 | 0.07 | 0.03 |
| Smaller Size | 6 | 106 | 115 | -24.90 | 3.77 | 3.07 | 1.69 | -0.85 | 0.03 | 5.95 | 5.83 | 0.23 | 0.16 |
| State Sphere |  |  |  |  |  |  |  |  |  |  |  |  |  |
| Larger Size | 10 | 7694 | 7790 | -7.15 | 0.73 | 1.05 | 0.11 | -0.43 | 0.03 | 4.24 | 2.09 | 0.08 | 0.04 |
| Smaller Size | 14 | 360 | 401 | -21.73 | 3.58 | 3.78 | 0.42 | -0.72 | 0.03 | 8.03 | 5.39 | 0.19 | 0.08 |
| **Age Group** |  |  |  |  |  |  |  |  |  |  |  |  |  |
| Before 1986 |  |  |  |  |  |  |  |  |  |  |  |  |  |
| Larger Size | 5 | 2116 | 2345 | -37.71 | 5.29 | 1.61 | 0.55 | -0.84 | 0.07 | 6.12 | 3.37 | 0.08 | 0.05 |
| Smaller Size | 5 | 163 | 192 | -38.12 | 4.84 | 3.08 | 0.75 | -0.92 | 0.04 | 11.05 | 9.23 | 0.33 | 0.11 |
| Between 1986-1996 |  |  |  |  |  |  |  |  |  |  |  |  |  |
| Larger Size | 3 | 754 | 815 | -32.38 | 2.31 | 1.34 | 0.22 | -0.84 | 0.04 | 2.27 | 1.25 | 0.14 | 0.06 |
| Smaller Size | 5 | 50 | 64 | -34.52 | 10.71 | 5.95 | 0.89 | -0.64 | 0.11 | 14.15 | 10.36 | 0.22 | 0.14 |
| Between 1996-2002 |  |  |  |  |  |  |  |  |  |  |  |  |  |
| Larger Size | 9 | 9429 | 9695 | 0.54 | 1.31 | 0.50 | 0.09 | -0.43 | 0.02 | 3.99 | 2.61 | 0.02 | 0.01 |
| Smaller Size | 6 | 100 | 107 | -7.48 | 3.02 | 2.72 | 0.25 | -0.38 | 0.06 | 6.07 | 4.65 | 0.03 | 0.03 |
| Between 2002-2008 |  |  |  |  |  |  |  |  |  |  |  |  |  |
| Larger Size | 2 | 280 | 281 | -4.13 | 1.27 | 1.53 | 0.06 | -0.50 | 0.02 | 3.58 | 1.72 | 0.03 | 0.02 |
| Smaller Size | 4 | 153 | 153 | -7.78 | 1.40 | 1.31 | 0.17 | -0.87 | 0.02 | 2.01 | 1.78 | 0.06 | 0.06 |

S.U. - sampling units (On – average number of S. U. on support); ATT – Absolute Effect, ATT% - Relative Effect; S.E - Standard Error; Mean - Mean for the 15 Best Models; Std. Dev. - Standard Deviation for the 15 Best Models, P. R^2^ – Pseudo R^2^. * No data.

**Table S12** – (continuation)

| **Groups/subgroups** |  | **Wilcoxon Paired Test** | | |
| --- | --- | --- | --- | --- |
|  | **ATT** | | **ATT%** | |
|  | **Z** | ***p*** | **Z** | ***p*** |
| **All PAs** |  |  |  |  |
| Larger Size | - | - | - | - |
| Smaller Size | 4.334 | <0.001 | 4.666 | <0.001 |
| **Restriction Group** |  |  |  |  |
| Strictly Protected |  |  |  |  |
| Larger Size | - | - | - | - |
| Smaller Size | -4.666 | <0.001 | -4.63 | <0.001 |
| Sustainable Use |  |  |  |  |
| Larger Size | - | - | - | - |
| Smaller Size | 3.173 | 0.002 | 4.666 | <0.001 |
| **Government Sphere Group** |  |  |  |  |
| Federal Sphere |  |  |  |  |
| Larger Size | - | - | - | - |
| Smaller Size | 3.173 | 0.002 | 4.666 | <0.001 |
| State Sphere |  |  |  |  |
| Larger Size | - | - | - | - |
| Smaller Size | 4.666 | <0.001 | 4.666 | <0.001 |
| **Age Group** |  |  |  |  |
| Before 1986 |  |  |  |  |
| Larger Size | - | - | - | - |
| Smaller Size | 0.062 | 0.95 | 2.924 | 0.004 |
| Between 1986-1996 |  |  |  |  |
| Larger Size | - | - | - | - |
| Smaller Size | 0.809 | 0.419 | -4.42 | <0.001 |
| Between 1996-2002 |  |  |  |  |
| Larger Size | - | - | - | - |
| Smaller Size | 4.666 | <0.001 | -1.8 | 0.071 |
| Between 2002-2008 |  |  |  |  |
| Larger Size | - | - | - | - |
| Smaller Size | 4.417 | <0.001 | 4.666 | <0.001 |
