# Supplementary material for: The Role of Protected Areas in the Avoidance of Anthropogenic Conversion in a High Pressure Region: A Matching Method Analysis in the Core Region of the Brazilian Cerrado
Source: PLoS One. 2015 Jul 29;10(7):e0132582. doi: 10.1371/journal.pone.0132582 (PMC4519267; doi:10.1371/journal.pone.0132582)
Supplement: S13 Table — (DOCX) [file pone.0132582.s015.docx]

**Table S13 –** Results for the age groups with respect to restriction, government sphere, and size subgroups.

| **Groups/subgroups** | **PA Units** | **S.U.** | | **ATT** | | **S.E.** | | **ATT%** | | **Bias** | | **P. R²** | |
| --- | --- | --- | --- | --- | --- | --- | --- | --- | --- | --- | --- | --- | --- |
|  |  |  |  |  |  |  |  |  |  |  |  |  |  |
|  |  | **On** | **Total** | **Mean** | **Std. Dev.** | **Mean** | **Std. Dev.** | **Mean** | **Std. Dev.** | **Mean** | **Std. Dev.** | **Mean** | **Std. Dev.** |
| **All PAs** |  |  |  |  |  |  |  |  |  |  |  |  |  |
| Before 1986 | 10 | 2279 | 2537 | -37.83 | 4.88 | 1.57 | 0.55 | -0.84 | 0.07 | 6.22 | 3.31 | 0.05 | 0.03 |
| Between 1986-1996 | 8 | 804 | 879 | -32.26 | 2.35 | 1.35 | 0.22 | -0.82 | 0.05 | 3.06 | 1.67 | 0.06 | 0.04 |
| Between 1996-2002 | 15 | 9530 | 9802 | 0.44 | 1.30 | 0.50 | 0.09 | -0.43 | 0.02 | 3.99 | 2.58 | 0.02 | 0.01 |
| Between 2002-2008 | 6 | 432 | 434 | -5.37 | 0.60 | 1.11 | 0.04 | -0.63 | 0.01 | 2.59 | 1.38 | 0.01 | 0.01 |
| **Restriction Group** |  |  |  |  |  |  |  |  |  |  |  |  |  |
| Strictly Protected |  |  |  |  |  |  |  |  |  |  |  |  |  |
| Before 1986 | 5 | 2032 | 2172 | -43.16 | 2.48 | 1.4 | 0.52 | -0.95 | 0.02 | 5.21 | 3.21 | 0.03 | 0.02 |
| Between 1986-1996 | 4 | 504 | 511 | -36.75 | 2.84 | 1.45 | 0.34 | -0.95 | 0.02 | 1.68 | 1.15 | 0.03 | 0.05 |
| Between 1996-2002 | 4 | 39 | 42 | -11.58 | 2.69 | 2.87 | 0.20 | -0.59 | 0.07 | 5.61 | 3.64 | 0.05 | 0.06 |
| Between 2002-2008 | 2 | 190 | 192 | 0.25 | 1.16 | 2.21 | 0.10 | -0.31 | 0.02 | 4.55 | 2.40 | 0.10 | 0.07 |
| Sustainable Use |  |  |  |  |  |  |  |  |  |  |  |  |  |
| Before 1986 | 5 | 246 | 365 | 11.78 | 9.63 | 10.42 | 10.3 | 0.21 | 0.15 | 19.03 | 17.82 | 0.85 | 0.24 |
| Between 1986-1996 | 4 | 301 | 368 | -24.98 | 5.52 | 2.18 | 0.27 | -0.62 | 0.07 | 5.00 | 3.11 | 0.34 | 0.06 |
| Between 1996-2002 | 11 | 9491 | 9760 | 0.50 | 1.31 | 0.50 | 0.09 | -0.43 | 0.02 | 3.99 | 2.60 | 0.02 | 0.01 |
| Between 2002-2008 | 4 | 242 | 242 | -9.84 | 0.72 | 0.68 | 0.06 | -0.88 | 0.01 | 1.54 | 1.36 | 0.03 | 0.04 |
| **Government Sphere Group** |  |  |  |  |  |  |  |  |  |  |  |  |  |
| Federal Sphere |  |  |  |  |  |  |  |  |  |  |  |  |  |
| Before 1986 | 7 | 2121 | 2352 | -37.77 | 5.22 | 1.61 | 0.55 | -0.84 | 0.07 | 6.12 | 3.38 | 0.08 | 0.05 |
| Between 1986-1996* | - | - | - | - | - | - | - | - | - | - | - | - | - |
| Between 1996-2002 | 5 | 2673 | 2925 | -4.81 | 2.68 | 0.79 | 0.12 | -0.54 | 0.06 | 3.77 | 2.04 | 0.04 | 0.03 |
| Between 2002-2008 | 3 | 184 | 184 | -10.46 | 1.24 | 0.85 | 0.07 | -0.86 | 0.02 | 1.22 | 1.10 | 0.03 | 0.04 |

S.U. - sampling units (On – average number of S. U. on support); ATT – Absolute Effect, ATT% - Relative Effect; S.E - Standard Error; Mean - Mean for the 15 Best Models; Std. Dev. - Standard Deviation for the 15 Best Models, P. R^2^ – Pseudo R^2^. * No data.

**Table S13** – (continuation)

| **Groups/subgroups** | **PA Units** | **S.U.** | | **ATT** | | **S.E.** | | **ATT%** | | **Bias** | | **P. R²** | |
| --- | --- | --- | --- | --- | --- | --- | --- | --- | --- | --- | --- | --- | --- |
|  |  |  |  |  |  |  |  |  |  |  |  |  |  |
|  |  | **On** | **Total** | **Mean** | **Std. Dev.** | **Mean** | **Std. Dev.** | **Mean** | **Std. Dev.** | **Mean** | **Std. Dev.** | **Mean** | **Std. Dev.** |
| State Sphere |  |  |  |  |  |  |  |  |  |  |  |  |  |
| Before 1986 | 3 | 158 | 185 | -37.6 | 4.71 | 3.08 | 0.72 | -0.92 | 0.04 | 10.91 | 9.06 | 0.35 | 0.14 |
| Between 1986-1996 | 8 | 804 | 879 | -32.26 | 2.35 | 1.35 | 0.22 | -0.82 | 0.05 | 3.06 | 1.67 | 0.06 | 0.04 |
| Between 1996-2002 | 10 | 6845 | 6877 | 2.40 | 0.84 | 0.56 | 0.10 | -0.39 | 0.03 | 4.90 | 2.95 | 0.03 | 0.02 |
| Between 2002-2008 | 3 | 248 | 250 | -1.68 | 0.95 | 1.75 | 0.07 | -0.46 | 0.02 | 3.86 | 1.85 | 0.02 | 0.02 |
| **Size Group** |  |  |  |  |  |  |  |  |  |  |  |  |  |
| Larger Size |  |  |  |  |  |  |  |  |  |  |  |  |  |
| Before 1986 | 5 | 2116 | 2345 | -37.71 | 5.29 | 1.61 | 0.55 | -0.84 | 0.07 | 6.12 | 3.37 | 0.08 | 0.05 |
| Between 1986-1996 | 3 | 754 | 815 | -32.38 | 2.31 | 1.34 | 0.22 | -0.84 | 0.04 | 2.27 | 1.25 | 0.14 | 0.06 |
| Between 1996-2002 | 9 | 9429 | 9695 | 0.54 | 1.31 | 0.50 | 0.09 | -0.43 | 0.02 | 3.99 | 2.61 | 0.02 | 0.01 |
| Between 2002-2008 | 2 | 280 | 281 | -4.13 | 1.27 | 1.53 | 0.06 | -0.50 | 0.02 | 3.58 | 1.72 | 0.03 | 0.02 |
| Smaller Size |  |  |  |  |  |  |  |  |  |  |  |  |  |
| Before 1986 | 5 | 163 | 192 | -38.12 | 4.84 | 3.08 | 0.75 | -0.92 | 0.04 | 11.05 | 9.23 | 0.33 | 0.11 |
| Between 1986-1996 | 5 | 50 | 64 | -34.52 | 10.71 | 5.95 | 0.89 | -0.64 | 0.11 | 14.15 | 10.36 | 0.22 | 0.14 |
| Between 1996-2002 | 6 | 100 | 107 | -7.48 | 3.02 | 2.72 | 0.25 | -0.38 | 0.06 | 6.07 | 4.65 | 0.03 | 0.03 |
| Between 2002-2008 | 4 | 153 | 153 | -7.78 | 1.40 | 1.31 | 0.17 | -0.87 | 0.02 | 2.01 | 1.78 | 0.06 | 0.06 |

S.U. - sampling units (On – average number of S. U. on support); ATT – Absolute Effect, ATT% - Relative Effect; S.E - Standard Error; Mean - Mean for the 15 Best Models; Std. Dev. - Standard Deviation for the 15 Best Models, P. R^2^ – Pseudo R^2^. * No data.

**Table S13** – (continuation)

| **Groups/subgroups** | **Wilcoxon Paired Test** | | | | | | | | | | | |
| --- | --- | --- | --- | --- | --- | --- | --- | --- | --- | --- | --- | --- |
|  | **ATT** | | | | | | **ATT%** | | | | | |
|  | **Z** | ***p*** | **Z** | ***p*** | **Z** | ***p*** | **Z** | ***p*** | **Z** | ***p*** | **Z** | ***p*** |
| **All PAs** | Before 1986 | | Btw. 1986-1996 | | Btw. 1996-2002 | | Before 1986 | | Btw. 1986-1996 | | Btw. 1996-2002 | |
| Before 1986 | - | - | - | - | - | - |  | - | - | - | - | - |
| Between 1986-1996 | -3.256 | 0.001 | - | - | - | - | -0.518 | 0.604 | - | - | - | - |
| Between 1996-2002 | -4.666 | <0.001 | -4.7 | <0.001 | - | - | -4.666 | <0.001 | -4.666 | <0.001 | - | - |
| Between 2002-2008 | -4.666 | <0.001 | -4.7 | <0.001 | 4.666 | <0.001 | -4.666 | <0.001 | -4.666 | <0.001 | 4.666 | <0.001 |
| **Restriction Group** |  |  |  |  |  |  |  |  |  |  |  |  |
| Strictly Protected |  |  |  |  |  |  |  |  |  |  |  |  |
| Before 1986 | - | - | - | - | - | - |  | - | - | - | - | - |
| Between 1986-1996 | -4.334 | <0.001 | - | - | - | - | -0.684 | 0.494 | - | - | - | - |
| Between 1996-2002 | -4.666 | <0.001 | -4.7 | <0.001 | - | - | -4.666 | <0.001 | -4.666 | <0.001 | - | - |
| Between 2002-2008 | -4.666 | <0.001 | -4.7 | <0.001 | -4.666 | <0.001 | -4.666 | <0.001 | -4.666 | <0.001 | -4.666 | <0.001 |
| Sustainable Use |  |  |  |  |  |  |  |  |  |  |  |  |
| Before 1986 | - | - | - | - | - | - |  | - | - | - | - | - |
| Between 1986-1996 | 4.666 | <0.001 | - | - | - | - | 4.666 | <0.001 | - | - | - | - |
| Between 1996-2002 | 3.38 | 0.001 | -4.7 | <0.001 | - | - | 4.666 | <0.001 | -4.666 | <0.001 | - | - |
| Between 2002-2008 | 4.666 | <0.001 | -4.7 | <0.001 | 4.666 | <0.001 | 4.666 | <0.001 | 4.666 | <0.001 | 4.666 | <0.001 |
| **Government Sphere Group** |  |  |  |  |  |  |  |  |  |  |  |  |
| Federal Sphere |  |  |  |  |  |  |  |  |  |  |  |  |
| Before 1986 | - | - | - | - | - | - | - | - | - | - | - | - |
| Between 1986-1996* | - | - | - | - | - | - | - | - | - | - | - | - |
| Between 1996-2002 | -4.666 | <0.001 | - | - | - | - | -4.666 | <0.001 | - | - | - | - |
| Between 2002-2008 | -4.666 | <0.001 | - | - | 4.376 | <0.001 | 2.053 | 0.040 | - | - | 4.666 | <0.001 |

ATT – Absolute Effect, ATT% - Relative Effect; S.E - Standard Error; * No data.

**Table S13** – (continuation)

| **Groups/subgroups** | **Wilcoxon Paired Test** | | | | | | | | | | | |
| --- | --- | --- | --- | --- | --- | --- | --- | --- | --- | --- | --- | --- |
|  | **ATT** | | | | | | **ATT%** | | | | | |
|  | **Z** | ***p*** | **Z** | ***p*** | **Z** | ***p*** | **Z** | ***p*** | **Z** | ***p*** | **Z** | ***p*** |
| State Sphere | Before 1986 | | Btw. 1986-1996 | | Btw. 1996-2002 | | Before 1986 | | Btw. 1986-1996 | | Btw. 1996-2002 | |
| Before 1986 | - | - | - | - | - | - |  | - | - | - | - | - |
| Between 1986-1996 | -3.173 | 0.002 | - | - | - | - | -4.127 | <0.001 | - | - | - | - |
| Between 1996-2002 | -4.666 | <0.001 | -4.7 | <0.001 | - | - | -4.666 | <0.001 | -4.666 | <0.001 | - | - |
| Between 2002-2008 | -4.666 | <0.001 | -4.7 | <0.001 | 4.666 | <0.001 | -4.666 | <0.001 | -4.666 | <0.001 | 4.666 | <0.001 |
| **Size Group** |  |  |  |  |  |  |  |  |  |  |  |  |
| Larger Size |  |  |  |  |  |  |  |  |  |  |  |  |
| Before 1986 | - | - | - | - | - | - |  | - | - | - | - | - |
| Between 1986-1996 | -3.173 | 0.002 | - | - | - | - | 0.518 | 0.604 | - | - | - | - |
| Between 1996-2002 | -4.666 | <0.001 | -4.7 | <0.001 | - | - | -4.666 | <0.001 | -4.666 | <0.001 | - | - |
| Between 2002-2008 | -4.666 | <0.001 | -4.7 | <0.001 | 4.625 | <0.001 | -4.666 | <0.001 | -4.666 | <0.001 | 4.583 | <0.001 |
| Smaller Size |  |  |  |  |  |  |  |  |  |  |  |  |
| Before 1986 | - | - | - | - | - | - |  | - | - | - | - | - |
| Between 1986-1996 | -1.182 | 0.240 | - | - | - | - | -4.666 | <0.001 | - | - | - | - |
| Between 1996-2002 | -4.666 | <0.001 | -4.7 | <0.001 | - | - | -4.666 | <0.001 | -4.666 | <0.001 | - | - |
| Between 2002-2008 | -4.666 | <0.001 | -4.7 | <0.001 | 0.27 | 0.788 | -4.086 | <0.001 | 4.666 | <0.001 | 4.666 | <0.001 |

ATT – Absolute Effect, ATT% - Relative Effect; S.E - Standard Error; * No data.
